# Chronic Aripiprazole and Trazodone Polypharmacy Effects on Systemic and Brain Cholesterol Biosynthesis

**DOI:** 10.3390/biom13091321

**Published:** 2023-08-28

**Authors:** Zeljka Korade, Allison Anderson, Marta Balog, Keri A. Tallman, Ned A. Porter, Karoly Mirnics

**Affiliations:** 1 Department of Pediatrics, College of Medicine, University of Nebraska Medical Center, Omaha, NE 68198, USA; zeljka.korade@unmc.edu; 2 Department of Biochemistry and Molecular Biology, College of Medicine, University of Nebraska Medical Center, Omaha, NE 68198, USA; 3 Munroe-Meyer Institute for Genetics and Rehabilitation, University of Nebraska Medical Center, Omaha, NE 68105, USA; allison.anderson@unmc.edu; 4 Department of Medical Biology and Genetics, Faculty of Medicine, Josip Juraj Strossmayer University of Osijek, 31000 Osijek, Croatia; mbalog@volunteer.unmc.edu; 5 Department of Chemistry, Vanderbilt University, Nashville, TN 37240, USA; keri.a.tallman@vanderbilt.edu (K.A.T.); n.porter@vanderbilt.edu (N.A.P.)

**Keywords:** trazodone (TRZ), aripiprazole (ARI), 7-dehydrocholesterol, desmosterol, DHCR7, IBA1

## Abstract

The concurrent use of several medications is a common practice in the treatment of complex psychiatric conditions. One such commonly used combination is aripiprazole (ARI), an antipsychotic, and trazodone (TRZ), an antidepressant. In addition to their effects on dopamine and serotonin systems, both of these compounds are inhibitors of the 7-dehydrocholesterol reductase (DHCR7) enzyme. To evaluate the systemic and nervous system distribution of ARI and TRZ and their effects on cholesterol biosynthesis, adult mice were treated with both ARI and TRZ for 21 days. The parent drugs, their metabolites, and sterols were analyzed in the brain and various organs of mice using LC-MS/MS. The analyses revealed that ARI, TRZ, and their metabolites were readily detectable in the brain and organs, leading to changes in the sterol profile. The levels of medications, their metabolites, and sterols differed across tissues with notable sex differences. Female mice showed higher turnover of ARI and more cholesterol clearance in the brain, with several post-lanosterol intermediates significantly altered. In addition to interfering with sterol biosynthesis, ARI and TRZ exposure led to decreased ionized calcium-binding adaptor molecule 1 (IBA1) and increased DHCR7 protein expression in the cortex. Changes in sterol profile have been also identified in the spleen, liver, and serum, underscoring the systemic effect of ARI and TRZ on sterol biosynthesis. Long-term use of concurrent ARI and TRZ warrants further studies to fully evaluate the lasting consequences of altered sterol biosynthesis on the whole body.

## 1. Introduction

Polypharmacy is a common practice especially when treating complex psychiatric conditions [1,2,3,4,5,6]. Based on the analysis of the Truven Health Market-Scan Database, among patients taking any prescription drug, half were exposed to two or more drugs, with 5% of patients being exposed to eight or more medications [1]. One of the frequent combinations used is the antipsychotic ARI and the anti-depressant TRZ. ARI is a partial agonist of dopamine D2 receptors, an antagonist of serotonin 2A receptors, and an agonist of serotonin 1A receptors [7,8]. TRZ inhibits the reuptake of serotonin and blocks the histamine and alpha-1-adrenergic receptors [9,10]. TRZ at a low dose has a sedative effect through antagonism of the 5HT-2A, H1, and alpha-1-adrenergic receptors [11,12]. The full spectrum of the TRZ mechanism of action is not fully understood [12].

Studies in rodents have shown that both ARI and TRZ affect brain tissue through unique signaling pathways and their effects are not always directly related to their binding on dopaminergic and serotonergic receptors [13,14,15,16,17,18,19,20,21,22]. ARI has been shown to have neuroprotective effects in stroke recovery by promoting dopaminergic survival and neuroprotective effects in a mouse model of Alzheimer’s Disease [23,24]. While many studies using rodent models have shown the therapeutic effects of ARI and TRZ in different pathological conditions, there are limited reports describing their biochemical effects in healthy mice.

A less well-known effect of both ARI and TRZ is the inhibition of DHCR7, a critical enzyme in cholesterol biosynthesis [13,25,26]. Our developmental studies in various in vitro and in vivo models have shown that ARI and TRZ, through the inhibition of DHCR7 enzyme, increase 7-DHC and 7-DHD, and decrease desmosterol and cholesterol [26,27]. These and other studies revealed that altering the cholesterol biosynthesis pathway has profound impacts on membrane properties, cell signaling, neurotransmitters, and neuronal morphology [28,29,30,31,32]. 

This study was designed to assess systemic and brain tissue distribution of ARI and TRZ in adult mice after prolonged treatment and to ascertain their combined effect on the cholesterol biosynthesis pathway. We found that a combination of these two medications altered cholesterol biosynthesis in both the central nervous system and non-neuronal tissues. We also observed that the ARI+TRZ combination led to the upregulation of DHCR7 protein and decreased protein expression of the microglial marker, IBA1.

## 2. Materials and Methods

### 2.1. Chemicals

Chemicals were purchased from Sigma-Aldrich Co. (St. Louis, MO, USA). HPLC grade solvents were purchased from Thermo Fisher Scientific Inc. (Waltham, MA, USA). TRZ and ARI were obtained from Selleckchem (Radnor, PA, USA) and dissolved in a sterile DMSO solution for the experiments. All natural and isotopically labeled sterol standards were described previously [33] and are available from Kerafast, Inc. (Boston, MA, USA). 

### 2.2. Mouse Model of Aripiprazole and Trazodone Exposure

Adult male and female C57Bl/6J stock #000664 mice, 3 months old, were purchased from Jackson Laboratories. Mice were housed under a 12 h light-dark cycle at constant temperature (25 °C) and humidity with *ad libitum* access to food (Teklad LM-485 Mouse/Rat Sterilizable Diet 7012) and water in Comparative Medicine at the University of Nebraska Medical Center (UNMC), Omaha, NE. The total number of adult mice was 32, and they were assigned to either the polypharmacy group (N = 17; 10 males and 7 females) or the control group (N = 15; 8 males and 7 females). Mice received intraperitoneal injections of the drug combination ARI (2.5 mg/kg) and TRZ (10 mg/kg) or vehicle (VEH), (5% DMSO in saline), with the exposure at 8.00 a.m. daily, for 21 days. In humans, TRZ (Desyrel) is given at a starting dose of 150 mg/day; and may be increased by 50 mg per day every 3 to 4 days to a maximum dose of 400 mg per day for outpatient use. For the treatment of insomnia, TRZ is given at a starting dose of 50 mg/day. The human dose of 50 mg/day translates to the animal equivalent dose of 10 mg/kg [34]. Based on these calculations and the literature data, we used ARI (Abilify) at 2.5 mg/kg in our mouse experiments (which corresponds to one 10–15 mg ARI tablet per day in humans). The range of common doses in humans is 2–30 mg/day. During the combination treatment over the 21 days of the experiment, sensitivity to the drug treatment differed for male and female mice (Figure S1A–C and Table S1). Males receiving A+T underwent an average change of −0.62 ± 0.169 g, compared with a 0.313 ± 0.220 g gain for those receiving VEH, a statistically significant difference (*p* = 0.0035). In contrast, the changes in body weight for female mice receiving either A+T (0.157 ± 0.545 g) or VEH (VEH −0.371 ± 0.208 g) were not significantly different (*p* = 0.383). All experimental procedures were approved by the University of Nebraska Medical Center’s Institutional Animal Care and Use Committee and followed guidelines in the Guide for the Care and Use of Laboratory Animals of the National Institute of Health.

### 2.3. Tissue Collection and Preparation for Sterol and Medications Measurements

Mice were euthanized with isoflurane overdose (Forane^®^ isofluranum, Abbott Laboratories Ltd.; Lake Bluff, IL, USA) two hours after they received their last injection on day 21. Brains from mice for biochemical analyses were dissected and brain regions were frozen in pre-chilled methyl-butane and stored at −80 °C. Frozen samples were sonicated in ice-cold PBS containing butylated hydroxytoluene (BHT) and triphenylphosphine (PPh_3_). The aliquots of homogenized tissue were used for sterol extraction, protein, ARI, TRZ, and metabolite measurements. The protein was measured using BCA assay (Pierce^TM^ BCA Protein Assay Kit, ThermoFisher Scientific, Waltham, MA, USA), reading absorbance at 562 nm using a Spectromax Plus 384 (Molecular Devices). Sterols were extracted using Folch solution as described previously [26]. ARI, TRZ, and their metabolites were extracted using methyl tert-butyl ether and ammonium hydroxide as described previously [13,25]. Sterols from tissues were normalized to protein measurements and expressed as nmol/mg protein. Sterols from serum were expressed as nmol/mL serum. ARI, TRZ, and their metabolite levels in tissues were normalized to protein measurements and expressed as ng/mg protein and in serum as ng/mL serum. 

### 2.4. (N,N-Dimethylglycyl) DMG Ester Derivatization Method for Sterol Measurements and LC-MS/MS Analysis

Derivatizing reagent was freshly prepared with 2-methyl-6-nitrobenzoic anhydride (20 mg), *N*,*N*-dimethylglycine (14 mg), DMAP (6 mg), and Et_3_N (0.1 mL) in anhydrous CHCl_3_ (0.9 mL). Derivatizing reagent (100 μL) was added to each sample and allowed to react at room temperature for 30 min. The samples were dried under vacuum and subsequently dissolved in MeOH (100 μL) for LC–MS/MS analysis. The samples were placed in an Acquity UPLC system coupled to a ThermoScientific TSQ Quantis mass spectrometer equipped with positive ion mode using an electrospray ionization (ESI) source. Then 10 μL were injected into the Agilent Poroshell EC-C18 column (10 cm × 2.1 mm, 1.9 μm) with CH_3_CN:MeOH:H_2_O, 70:25:5 (0.01% (v), formic acid, 1 mM NH_4_OAc) mobile phase at a column temperature of 40 °C. The flow rate was 400 μL/min for 11.5 min, then ramped to 600 μL/min at 11.6 min with a total run time of 23.1 min. MS parameters were optimized using DMG-Chol and were as follows: spray voltage at 4500 V, capillary temperature at 300 °C, auxiliary nitrogen gas pressure at 55 psi, and sheath gas pressure at 60 psi. Collision energy (CE) was optimized for each sterol and oxysterol under a collision gas pressure of 1.5 m Torr. The monitored transitions were as previously described [33]. Data were acquired with a Finnigan Xcalibur software package. Final sterol numbers are reported as nmol/mg total protein or nmol/mL serum.

### 2.5. Immunohistochemistry

Mice used for immunohistochemical analysis were anesthetized with isoflurane and transcardially perfused with saline followed by 4% paraformaldehyde. Brains were harvested and postfixed in 4% paraformaldehyde overnight at 4 °C. After fixation brains were incubated sequentially in 10%, 20%, and 30% sucrose solutions, each for 24 h at 4 °C. Next, brains were frozen and cut on a cryostat as 30 µm coronal sections. Floating sections were collected and processed for immunohistochemistry and then mounted on glass slides. To aid in visualizing brain structures, nuclei were counterstained with Hoechst dye (0.5 μg/mL final concentration, ThermoFisher Scientific, Waltham, MA, USA). The primary antibodies used include Doublecortin (DCX) (Cell Signaling, cat. No: 4604, host: rabbit, dilution used 1:800), and IBA1 (Cell Signaling, cat. No: 17198S, host: rabbit, dilution used 1:500). The secondary antibody used was donkey-anti-rabbit CY3 (Jackson Immuno Research, cat. No: 711-165-152, dilution used: 1:500). Sections were imaged with the EVOS microscope or ImageXpress Pico instrument using 4×, 10×, or 20× magnification.

### 2.6. Total RNA Extraction, cDNA Synthesis, and qPCR Analysis

RNA from adult mouse brains was isolated using RNeasy Mini Kit following the manufacturer’s protocol (Qiagen; Germantown, MD, USA). RNA concentration and quality were measured using a NanoDrop 2000 Spectrophotometer (Thermo Fisher Scientific). Following the manufacturer’s protocol, the first strand cDNA synthesis kit (Qiagen, Germantown, MD, USA) was used for reverse transcription of 500 ng total RNA. The cDNA was then diluted with nuclease-free water. Real-time PCR was performed on a StepOnePlus Real-Time PCR System (Applied Biosystems, Foster City, CA, USA) using SYBR green. Data collection was assisted by the StepOne Software v2.3 (Thermo Fisher Scientific). Samples were analyzed using the ∆∆C_t_ method [35].

### 2.7. Western Blotting

The cortex was lysed in Syn-PER^TM^ Synaptic Protein Extraction Reagent (ThermoFisher) supplemented with added Protease Inhibitor Cocktail and Phosphatase Inhibitor Cocktail. The protein concentration was determined using the Pierce^TM^ BCA Protein Assay Kit. For western blot analysis, 20 or 40 µg of total protein was loaded on NuPAGE^TM^ 4–12% Bis-Tris, 1.0 mm mini protein gels (Invitrogen) and electrophoretically transferred to PVDF membrane in ice-cold transfer buffer (Tris/Glycine with 20% methanol). The membrane was blocked with 5% dry milk in TBST (50 mM Tris pH 7.6 and 0.1% Igepal) for 30 min at room temperature. The blots were incubated overnight at 4°C with the primary antibodies. Primary antibodies: DHCR7 (Invitrogen, cat. No: PA5-48204, host: rabbit, dilution used 1:2000), IBA1 (Cell Signaling, cat. No: 17198S, host: rabbit, dilution used 1:1000); secondary antibody: HRP anti-rabbit (Cell Signaling, cat. No. 7074S, dilution used 1:3000). After three washes, the membranes were incubated with the secondary antibodies for 1 h at RT. After extensive washing, the blots were exposed to a chemiluminescence reagent (Radiance Q, Azure Biosystems AC2101) and visualized on an Azure 300 instrument (Azure Biosystems, Dublin, CA, USA). The immunoreactive signals were quantified using AzureSpot Pro and densitometric values for the proteins of interest were normalized using GAPDH (Cell Signaling, cat. No. 5174, host: rabbit, dilution used 1:30,000) and beta-actin (Cell Signaling, cat. No. 3700, host: mouse, dilution used 1:3000); secondary antibodies: HRP anti-mouse (cell Signaling, cat. No. 7076S, dilution used 1:3000), HRP anti-rabbit (Cell Signaling, cat. No. 7074S, dilution used 1:3000). Western blots were arranged by gender, in which each gel contained 10 samples (5 VEH and 5 ARI+TRZ males; and 5 VEH and 5 ARI+TRZ females; total 20 biological replicates). All samples were run in technical duplicates using a second set of gels. 

### 2.8. Statistical Analyses

Statistical analyses were performed using GraphPad Prism version 9 (GraphPad Software, Inc., La Jolla, CA, USA) for Windows and Microsoft Excel 2016. All data were analyzed by two-way analysis of variance (ANOVA) (treatment × sex). Data are presented as the mean ± standard error of the mean (SEM). Differences in means were tested using unpaired two-tailed *t*-tests, employing Welch’s correction when the variances between the two groups were significantly different. The *p*-values for statistically significant differences are highlighted in figure legends. Statistical significance was accepted when *p* < 0.05. 

## 3. Results

### 3.1. ARI, TRZ, and Metabolites Measurements in Serum and Tissues

In mice exposed to ARI+TRZ for 21 days the levels of ARI, TRZ, and their metabolites were measured in serum, liver, spleen, neocortex, and hippocampus (Figures S2 and S3; Tables S2 and S3). ARI and metabolites dehydroaripiprazole (d-ARI), 2,3-dichlorophenylpiperazine (2,3-DCPP), and TRZ and its metabolite, *meta*-chlorophenylpiperazine (*m*-CPP) (Figure S2 and Table S2), are readily detectable in all samples. The graphs and table show drug and metabolite levels in males and females separately. Serum and liver have the highest level of ARI when compared to spleen, cortex, and hippocampus. The d-ARI level is highest in the serum with similar lower levels in the liver and spleen and lowest levels in the cortex and hippocampus. The 2,3-DCPP levels are comparable across different organs with the highest levels in the serum. ARI is present at higher levels than any of its metabolites in all tissues, which reflects its long half-life. The turnover of ARI, expressed as the d-ARI to ARI ratio, varies across tissues and it is higher in females than males (Figure S3 and Table S3). The ratio of another ARI metabolite, 2,3-DCPP, (2,3-DCPP/ARI) in the cortex and hippocampus is about 10 times higher than in serum, liver, and spleen suggesting that 2,3-DCPP might take longer to clear from brain tissue. TRZ has a short half-life and is quickly metabolized so there are lower levels of TRZ than its metabolite in all tested tissues. However, the *m*-CPP to TRZ ratio differs across tissues but it is similar in females and males. The overall data reveal that ARI and TRZ are differently metabolized in specific organs and their metabolism differs between males and females. 

### 3.2. ARI+TRZ Polypharmacy Alters the Cholesterol Synthesis Pathway in Serum, Liver, and Spleen

**Figure 1:**
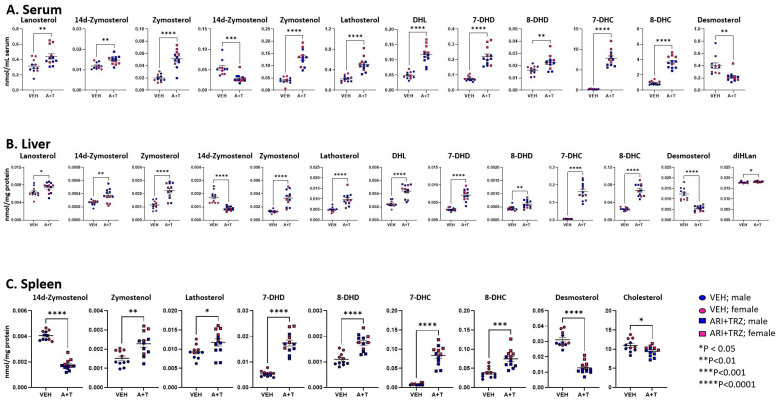
Levels of sterols in post-lanosterol synthesis pathway in serum (**A**), liver (**B**), and spleen (**C**). VEH injection (circles), ARI+TRZ exposure (squares), males (blue), and females (red). Two-tailed unpaired *t*-tests were used to determine significance. Only sterols that exhibit a significant difference in concentration are shown; the levels of all sterols are presented in Table S4.

Serum, liver, and spleen from the same mice were analyzed for sterol levels by the DMG method using LC-MS/MS. This method allows quantitative measurements of 14 sterols in the post-lanosterol cholesterol synthesis pathway and 7 oxysterols. The combined ARI+TRZ exposure led to altered levels of multiple post-lanosterol intermediates. In serum and liver, 12 and 13 sterols, and in the spleen, 9 sterols were changed in response to ARI+TRZ exposure (Figure 1, Table S4). 

Figure 1 denotes sterols that are significantly changed. Table S4 shows the mean ± SEM for all analyzed sterols and oxysterols across the three tested peripheral organs. Notably, in serum, liver, and spleen 10 sterol intermediates were increased, with only the levels of 14d-zymostenol and desmosterol decreased in all three tissues. While multiple intermediates in the post-lanosterol pathway were affected, the cholesterol level was significantly decreased only in the spleen. Additionally, we also observed several intriguing oxysterol changes. 

**Figure 2:**
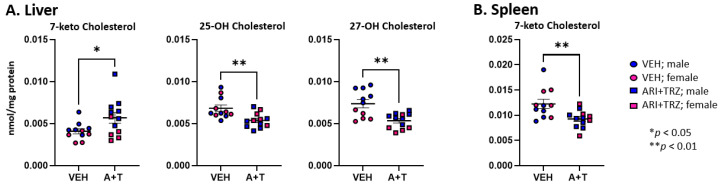
Cholesterol-derived oxysterols in liver and spleen. VEH injection (circles), ARI+TRZ treatment (squares), males (blue), and females (red). Two-tailed unpaired *t*-tests were used to determine significance. Only oxysterols that exhibit a significant difference in concentration are shown; the levels of all oxysterols are presented in Table S4.

Measurements of oxysterols in the liver (Figure 2) showed elevated levels of 7-ketocholesterol and decreased levels of 25-OH Chol and 27-OH Chol. In the spleen 7-keto Chol was decreased, most likely reflecting the overall decrease in cholesterol. There were no detectable oxysterol changes in serum. Among sterols and oxysterols, the most pronounced change is in the elevation of 7-DHC in ARI+TRZ exposed mice: it shows an approximately a 30-fold increase in the serum, a 25-fold increase in liver, and a 10-fold increase in spleen over untreated controls (Figure S4). 

We observed several notable sterol level differences between vehicle-treated control male and female mice (Figures S5–S7 and Table S5). Serum and spleen concentrations of 7-DHD, 7-DHC, 8-DHC, and beta-epoxycholesterol levels were higher in females than in males. In the liver, the same female mice had lower levels of 14dZyme, Des, Chol, Lath, 7-keto Chol, and 27-OH Chol. 

In summary, liver, serum, and spleen sterol intermediate levels of untreated adult mice show notable sex differences. In addition, various sterol levels show significant variations across the peripheral tissues under untreated control conditions. In contrast, ARI+TRZ polypharmacy exposure has a robust, broadly similar effect on sterol compounds in these three organs in both male and female mice. 

### 3.3. ARI+TRZ Polypharmacy Alters Cholesterol Synthesis Pathway in the Brain

**Figure 3:**
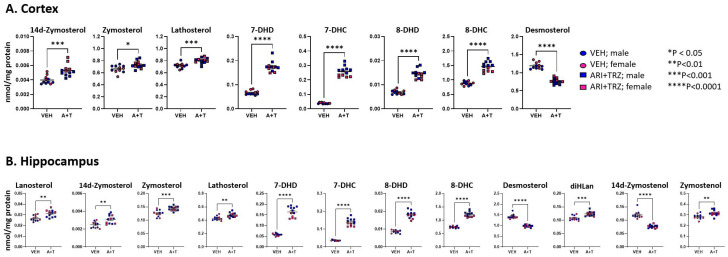
Levels of sterols in post-lanosterol synthesis pathway in cortex (**A**) and hippocampus (**B**). VEH injection (circles), ARI+TRZ treatment (squares), males (blue), and females (red). Two-tailed unpaired *t*-tests were used to determine significance. Only sterols that exhibit a significant difference in concentration are shown; the levels of all sterols are presented in Table S4.

Brain cholesterol synthesis is independent of systemic sterol biosynthesis. ARI and TRZ, in addition to their primary therapeutic mechanisms of action, have also an off-target effect of inhibiting DHCR7 in the brain [13,25,26]. We tested the levels of 14 sterols and 7 oxysterols in both cortex and hippocampus. We found that 8 sterols were changed in the cortex and 12 sterols were changed in the hippocampus of the ARI+TRZ exposed mice. Long-term ARI+TRZ exposure significantly elevated 7-DHC and 7-DHD, and decreased desmosterol both in the neocortex and hippocampus (Tables S4 and S5 and Figure 3). 

Similarly, as seen in the liver and spleen, ARI+TRZ exposure in the brain inhibited EBP and SC5D resulting in significantly elevated 14d-zymosterol, zymosterol, lathosterol, 8-DHC, and 8-DHD compared to the vehicle exposed group. 

Within brain regions, we also observed multiple sex differences in sterol levels in untreated and treated animals (Figures S8–S10 and Table S5). Neocortical sterols 14dzym, DHL, 7-DHD, Des, 24-OHChol, 25-OHChol, 27-OHChol, and beta-epoxycholesterol all showed higher levels in untreated females. 

### 3.4. Long-Term ARI+TRZ Polypharmacy Increases DHCR7 Enzyme Expression in the Brain

**Figure 4:**
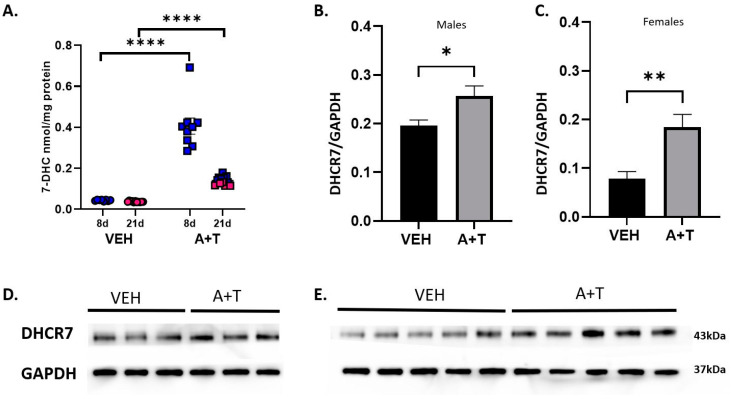
Levels of 7-DHC and DHCR7 enzyme. (**A**) 7-DHC levels in VEH (circles), ARI+TRZ (squares), males (blue), and females (red) during short-term and long-term exposures. (**B**,**C**) Quantification of western blots for DHCR7 in the cortex of male and female mice following VEH and ARI+TRZ exposure. DHCR7 protein band intensity of the western membrane was divided by the intensity of the GAPDH. (**D**) Example of one western blot for DHCR7 protein levels in male mouse cortices following long-term VEH and ARI+TRZ exposure. (**E**) An example of a western blot for DHCR7 protein in female mouse cortices following long-term VEH and ARI+TRZ exposure. * *p* < 0.05; ** *p* < 0.01; **** *p* < 0.0001 using two-tail *t*-tests.

Levels of sterol pathway intermediates can affect the expression of enzymes [36,37,38,39]. Since 7-DHC was the most increased metabolite as a result of ARI+TRZ polypharmacy, we decided to evaluate the protein levels of the DHCR7 enzyme. This line of investigation was further warranted by an unexpected finding. We found overall 7-DHC accumulation in long-term ARI+TRZ polypharmacy (21 days) was lower than observed in similar short-term treatment of 8 days [13] (Figure 4A). 

Thus, we hypothesized that we are observing an adaptation mechanism counteracting the deleteriously increased 7-DHC levels: upregulation of the DHCR7 enzyme that converts 7-DHC to cholesterol. Indeed, analyzing DHCR7 enzyme expression by western blotting revealed that long-term ARI+TRZ exposure increases the level of DHCR7 enzyme (Figure 4B–E) (males *p* = 0.0328; females *p* = 0.0073) which would in turn favor conversion of 7-DHC to cholesterol and explain the decreasing amount of 7-DHC. 

### 3.5. ARI+TRZ Polypharmacy Effects on Hippocampal Neurogenesis

Several studies report inconsistent data regarding the effects of ARI and TRZ on adult neurogenesis [40,41,42]. Doublecortin (DCX) is expressed in migrating neuroblasts and is used as a marker of adult neurogenesis [43]. To test if ARI+TRZ polypharmacy could facilitate or impede adult neurogenesis, we used qPCR to analyze total DCX mRNA. 

**Figure 5:**
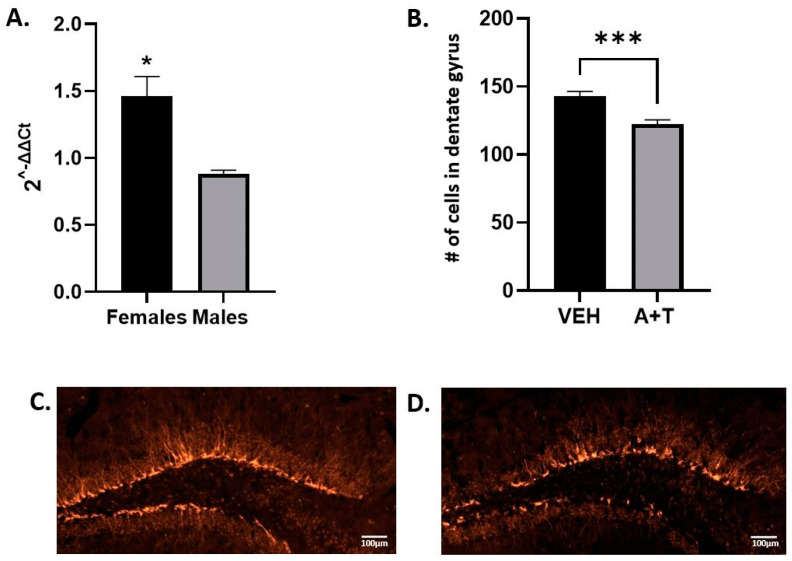
Adult neurogenesis in the hippocampus. (**A**) 2^ΔΔCt^ showing DCX mRNA expression in hippocampus (n = 7 ARI+TRZ males, n = 6 VEH males; *p* = 0.054; n = 5 ARI+TRZ females, n = 5 VEH females; *p* = 0.019). (**B**) The graph shows DCX-positive cell counts in the male dentate gyrus. Cells were counted in 20 matched sections in two VEH and two ARI+TRZ exposed mice; the graph shows the average number of cells per section. (**C**,**D**) Representative microscopy images of DCX immunohistochemistry detection in VEH and ARI+TRZ exposed mice in the dentate gyrus. * *p* < 0.05; *** *p* < 0.01 using two-tail *t*-tests.

The results show decreased DCX mRNA in males (2^−ΔΔCt^ mean ± SEM 0.88 ± 0.03, *p* = 0.05) and increased DCX in females (2^−ΔΔCt^ mean ± SEM 1.46 ± 0.15, *p* = 0.019) (Figure 5). A differential finding in males and females is not surprising because DCX is an X-linked gene [44]. In addition to qPCR, we did immunostaining of brain sections for DCX to quantify the intermediate neuronal progenitors. Figure 5 shows the representative images of brain sections from VEH and ARI+TRZ exposed mice. DCX showed cytosolic staining in an intracellular fluorescence pattern that was identical across the conditions. Quantification of neural progenitor cells revealed that in male mice ARI+TRZ decreased the number of DCX-positive cells (VEH = 143 vs. A+T = 122 *p* = 0.0007). DCX as measured with qPCR showed a similar differential expression pattern between ARI+TRZ and VEH male mice, although not a statistically significant difference.

### 3.6. ARI+TRZ Polypharmacy Decreases IBA1 Protein Levels in the Brain

ARI treatment in several rodent models led to decreased IBA1 protein expression [23,24,40]. IBA1 protein is specifically expressed in brain microglia and macrophages and is upregulated during their activation [45]. Activation of microglia commonly occurs in the early response of the CNS to a wide variety of pathological events [46,47]. To test if ARI+TRZ polypharmacy affects IBA1 expression we performed western blotting. 

**Figure 6:**
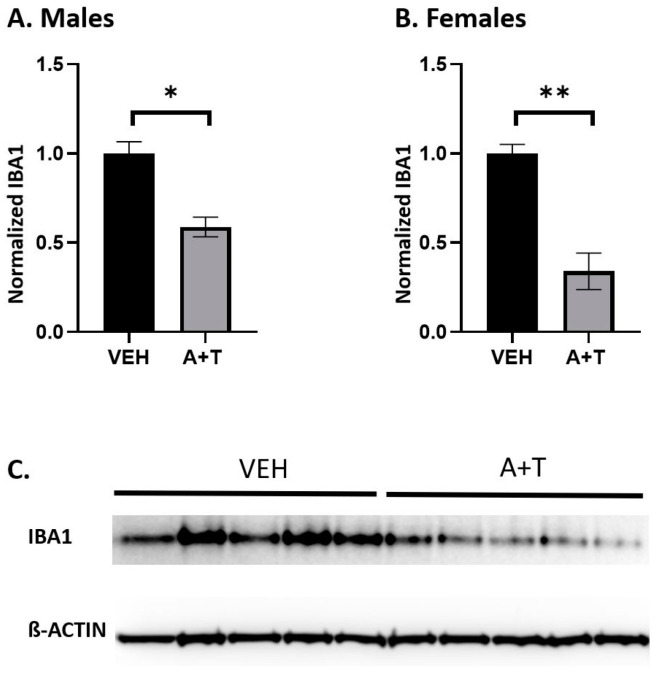
IBA1 protein is decreased in the cortex of both male and female mice. Quantification of western blots for IBA1 in male and female cortices following VEH and ARI+TRZ exposure. (**A**) Normalized IBA1 protein expression in males (* *p* = 0.0148; two-tail *t*-test). (**B**) Normalized IBA1 protein expression in females (** *p* < 0.01; two-tail *t*-test). (**C**) An example of IBA1 and ß-ACTIN western blotting in the male cortex (10 biological replicates).

We found that the levels of IBA1 protein were decreased in both males (*p* = 0.0148) and females (*p* < 0.01) exposed to ARI+TRZ, compared with VEH-exposed mice (Figure 6). 

## 4. Discussion

In summary, our studies revealed that (1) ARI+TRZ long-term polypharmacy leads to altered levels of many intermediates of the post-lanosterol pathway in both male and female mice. (2) This polypharmacy affects multiple organ systems of the body, with notable differences in drug distribution, drug metabolite levels, and sterol analyte levels depending on the biomaterial type. (3) Furthermore, this treatment triggers an adaptational response by elevating DHCR7 expression to counteract the DHCR7-inhibiting effects of ARI+TRZ. (4) ARI+TRZ long-term polypharmacy leads to decreased proliferation and reduction of neural progenitor cells in the hippocampi of male adult mice, and decreased expression of microglial marker IBA1 in the brain. (5) Baseline levels of sterol intermediates differ substantially across the different systemic and brain region specimens. (6) Finally, our data revealed sex-specific differences in baseline sterol analyte levels, underscoring a need for a separate assessment of male and female responses to medications that interfere with sterol biosynthesis. 

The effects of ARI+TRZ polypharmacy are complex, and they are very helpful in the treatment of psychiatric disorders [5,6]. Their mechanism of action is attributed to their receptor-mediated signaling [9], and their concurrent effects on sterol biosynthesis are routinely overlooked [13,25]. Furthermore, it is often assumed that their effect is limited to the brain, disregarding the biochemical changes that are occurring system-wide [25]. Yet, their effect on the systemic sterol biosynthesis has already been demonstrated. The first reports of strong 7-DHC elevation in the serum by ARI and TRZ, independently of *DHCR7* gene mutations, emerged more than a decade ago [48]. 

This all brings us to the critical question: Is 7-DHC elevation good or bad? We believe that this is fully context-dependent. It is well established that 7-DHC is the most oxidizable lipid known to date, with a propagation rate constant of 2160 (this is 200 times more than cholesterol and 10 times more than arachidonic acid) [49,50,51,52,53,54,55,56]. This highly reactive property of 7-DHC can be quite beneficial or seriously detrimental—depending on timing, location, and magnitude of change. During development, full or partial replacement of cholesterol with 7-DHC disrupts fundamental processes [57,58]. Elevation of 7-DHC by genetic disruption of the *DHCR7* gene leads to a severe intellectual and developmental disability known as Smith-Lemli-Opitz syndrome (SLOS) [59]. Similarly, increased levels of 7-DHC by many medications (including TRZ and ARI) lead to increased production of 7-DHC-derived oxysterols, and these reactive electrophiles impair cell viability, differentiation, and growth [60,61]. All the data to date would suggest that pronounced elevation of 7-DHC during development (through its oxysterols) is detrimental. In the field of cancer research, DHCR7 expression levels were found to be positively correlated with the infiltration of cancer-associated fibroblasts (CAFs) and myeloid-derived suppressor cells (MDSCs), and negatively correlated with anti-tumor immune cells in a majority of tumors [62]. For example, in pancreatic cancer, high *DHCR7* gene expression results in shorter survival of patients [63]. It seems that activating the Kandutsch-Russell pathway helps the cancer cells produce enough cholesterol to assemble new membranes, a requirement for accelerated proliferation [64].

Yet, it appears that in two other conditions and life stages, 7-DHC has beneficial effects. First, inhibiting DHCR7 protects mice from various viral infections [65,66]. Furthermore, ARI at low doses acts as an antimicrobial agent, inhibits biofilm formation, and impedes yeast-to-hyphal transition and flocculation [67]. Second, medications that increase 7-DHC levels protect from ferroptosis and reduce brain tissue damage in newborn rodent models of hypoxic-ischemic brains [68]. We believe that this beneficial effect is mediated by the ability of high 7-DHC levels to quench toxic free radicals, thus protecting the brain cells. In our experiment adult hippocampal neurogenesis was differentially affected by the ARI+TRZ polypharmacy, as expression of DCX was increased in females and decreased in males. Moreover, the microglial marker, IBA1 protein, was reduced in both male and female mice. 

The other important set of findings from our study is the systemic effects of psychotropic medication polypharmacy. The tissues we investigated have different sterol content, and they metabolize ARI and TRZ at different rates. Note that the levels of unesterified sterol intermediates and unesterified cholesterol within the brain are significantly higher than seen in the liver and spleen. In organs and the brain, the dominant intermediates belong to both the Bloch and Kandutsch-Russell pathways, confirming a previous study that both arms contribute to cellular cholesterol synthesis [69]. The effects of these medications on other tissues outside the brain are very likely and should be further investigated. 

Our study was set up to compare the systemic effects of polypharmacy on sterol biosynthesis. To fully appreciate the complexities of their actions, we obtained measurements of the two drugs, their metabolites, and their effect on sterol biosynthesis across four biomaterials. The majority of studies performed to date analyzed one or two of these aspects, limiting insights into the health consequences of the full set of post-lanosterol analytes. Lanosterol is the first steroidal precursor containing a double bond at C-24 and represents the branching point between the Bloch and the Kandutsch-Russell pathways. However, it seems that these two cholesterol biosynthesis paths are more didactical than functional. The recent studies of Mitsche et al. underscore this, revealing a dynamic, “hybrid pathway” (with different proportions contributed by Kandutsch-Russell and Bloch pathways), that is highly tissue type dependent [69]. The outcomes of our studies concur with and expand these findings.

Our study also highlights the remarkable differences between the male and female sterol homeostasis. The finding of elevated cholesterol-derived oxysterols in females, despite stable overall cholesterol levels, suggests baseline cholesterol turnover may be increased in females. Our findings agree with recently published work [70] showing a significantly higher baseline brain cholesterol clearance via CYP46A1 in women than in age-matched men. The observed male vs. female difference in 8-DHC can be attributed to the action of the X-linked EBP enzyme, while the origins of the other differences are unknown at the current time.

Finally, it is also notable that ARI+TRZ polypharmacy does not only affect 7-DHC levels but also multiple other post-lanosterol intermediates. We believe that these changes are adaptational, and DHCR7 inhibition and 7-DHC elevation trigger feedback loops, affecting many sterol intermediates with different biological roles. The accumulation of these precursors could have different roles in different tissues, which are mostly unknown to date. 

## 5. Conclusions

In conclusion, we believe that 7-DHC is a Janus molecule, with both beneficial and deleterious effects based on the local molecular environment. Furthermore, the male-female differences, disparities in sterol metabolism across organs and tissues, interconnected and adaptive sterol biochemical networks, and robust response to medications all underscore the fragility of the post-lanosterol biosynthetic pathway and the need for further study to inform safe and effective medication practices. 

## Data Availability

All data supporting reported results can be found in the Supplementary Materials.

## Supplementary Materials

The following supporting information can be downloaded at: https://www.mdpi.com/article/10.3390/biom13091321/s1, Figure S1: Mouse weights during ARI+TRZ exposure. (A) Average body weight of male and female mice at the end of 21-day exposure with ARI (2.5 mg/kg) + TRZ (10 mg/mg). Males VEH average weight (24.34 ± 0.07) vs. ARI+TRZ average weight (22.91 ± 0.96) *p* = 0.0041. Females VEH average weight (21.4 ± 0.99) vs. A+T average weight (20.27 ± 2.16) *p* = 0.2734. Sex differences for VEH *p* ≤ 0.0001 and A+T *p* = 0.0059. 2-way ANOVA shows that sex and treatment significantly influenced body weight (statistics shown in Table S1). (B) The change in weight (end of the experiment—beginning of the experiment) for each male (blue) mouse was graphed. The *t*-test analysis shows that males exposed to ARI+TRZ lost their weight during treatment. (C) The same comparison in female (red) mice did not show a difference between the control and treated groups. ** *p* < 0.01; **** *p* < 0.0001. Statistical details are in Table S1. Figure S2: Aripiprazole with its metabolites and trazodone with its metabolite in serum and organs. Average ARI, d-ARI, 2,3-DCPP, TRZ, and m-CPP in males (blue) and females (red) exposed to ARI+TRZ for 21 days with 2-way ANOVA. (A) Serum. (B) Liver. (C) Spleen. (D) Cortex. (E) Hippocampus. Statistical details are in Table S2. Figure S3: Comparison of ARI and TRZ turnover in males and females across tissues. (A) D-ARI/ARI ratio is higher in females than in males in all organs. The serum values did not reach statistical significance but showed the same trend as in other organs. (B) 2,3-DCPP/ARI ratio is higher in males than in females. The hippocampus values did not reach statistical significance but showed the same trend as in other organs. (C) m-CPP/TRZ ratio is similar in males and females across all organs. Statistical details are in Table S3. Figure S4: 7-DHC fold increase in response to ARI+TRZ exposure. Compared to the levels of 7-DHC in VEH-exposed mice, the levels of 7-DHC in ARI+TRZ-exposed mice were significantly increased in all analyzed tissues. Figure S5: Comparison of sterols and oxysterols between males and females within the serum. Graphs show levels of sterols and oxysterols in males (blue) and females (red) under control (circles) and experimental (squares) conditions. Two-tailed unpaired *t*-tests were used to determine significance. Figure S6: Comparison of sterols and oxysterols between males and females within the liver. Graphs show levels of sterols and oxysterols in males (blue) and females (red) under control (circles) and experimental (squares) conditions. Two-tailed unpaired *t*-tests were used to determine significance. Figure S7: Comparison of sterols and oxysterols between males and females within the spleen. Graphs show levels of sterols and oxysterols in males (blue) and females (red) under control (circles) and experimental (squares) conditions. Two-tailed unpaired *t*-tests were used to determine significance. Figure S8: Comparison of sterols and oxysterols between males and females within the cortex. Graphs show levels of sterols and oxysterols in males (blue) and females (red) under control (circles) and experimental (squares) conditions. Two-tailed unpaired *t*-tests were used to determine significance. Figure S9: Comparison of sterols and oxysterols between males and females within the hippocampus. Graphs show levels of sterols and oxysterols in males (blue) and females (red) under control (circles) and experimental (squares) conditions. Two-tailed unpaired t-tests were used to determine significance. Figure S10: Long-term ARI+TRZ inhibits multiple enzymes in the post-lanosterol cholesterol biosynthesis pathway within the cortex. The graphs are presented to coincide with the sterol biosynthesis showing Bloch and Kandutsch-Russell branches. Table S1: Mouse weights. Table S2: ARI, TRZ, and metabolites in serum and organs. Table S3: ARI and TRZ turnover in serum and organs. Table S4: Sterols and oxysterol levels (Mean ± SEM) in serum, and organs in VEH and ARI+TRZ exposed mice. Table S5: Sterols and oxysterol levels (Mean ± SEM) in serum and organs in male and female VEH and ARI+TRZ exposed mice.

## Abbreviations

7-DHC	7-dehydrocholesterol
ARI	aripiprazole
BHT	butylated hydroxytoluene
CHOL	cholesterol
DCX	doublecortin
DES	desmosterol
DHCEO	3β,5α-dihydroxycholest-7-en-6-one
Dhcr7 or DHCR7	7-dehydrocholesterol reductase
DHCR24	24-dehydrocholesterol reductase
IBA1	ionized calcium-binding adaptor molecule 1
LAN	lanosterol
SLOS	Smith-Lemli-Opitz syndrome
SRM	selective reaction monitoring
TRZ	trazodone
8-DHC	8-dehydrocholesterol
Lath	lathosterol
Zym	zymosterol
Zyme	zymostenol
7-DHD	7-dehydrodesmosterol
8-DHD	8-dehydrodesmosterol
14d-Zym	14d-zymosterol
DHL	dehydrolathosterol
14d-Zyme	14d-zymostenol
diHLan	dihydrolanosterol
7keto-Chol	7ketocholesterol
25OH-Chol	25-hydroxycholesterol
β epChol	beta epoxycholesterol
α epChol	alpha epoxycholesterol
DHCAO	DHCAO
24OH-Chol	24-hydroxycholesterol
27OH-Chol	27-hydroxycholesterol
d-ARI	dehydroaripiprazole
2,3-DCPP	2,3-dichlorophenylpiperazine
*m*-CPP	*meta*-chlorophenylpiperazine
