# Supplementary material for: Chronic Aripiprazole and Trazodone Polypharmacy Effects on Systemic and Brain Cholesterol Biosynthesis
Source: Biomolecules. 2023 Aug 28;13(9):1321. doi: 10.3390/biom13091321 (PMC10526910; doi:10.3390/biom13091321)
Supplement: Supplementary file 1 [file biomolecules-13-01321-s001.zip › biomolecules-2541146-SI.pdf]

Figure S1.

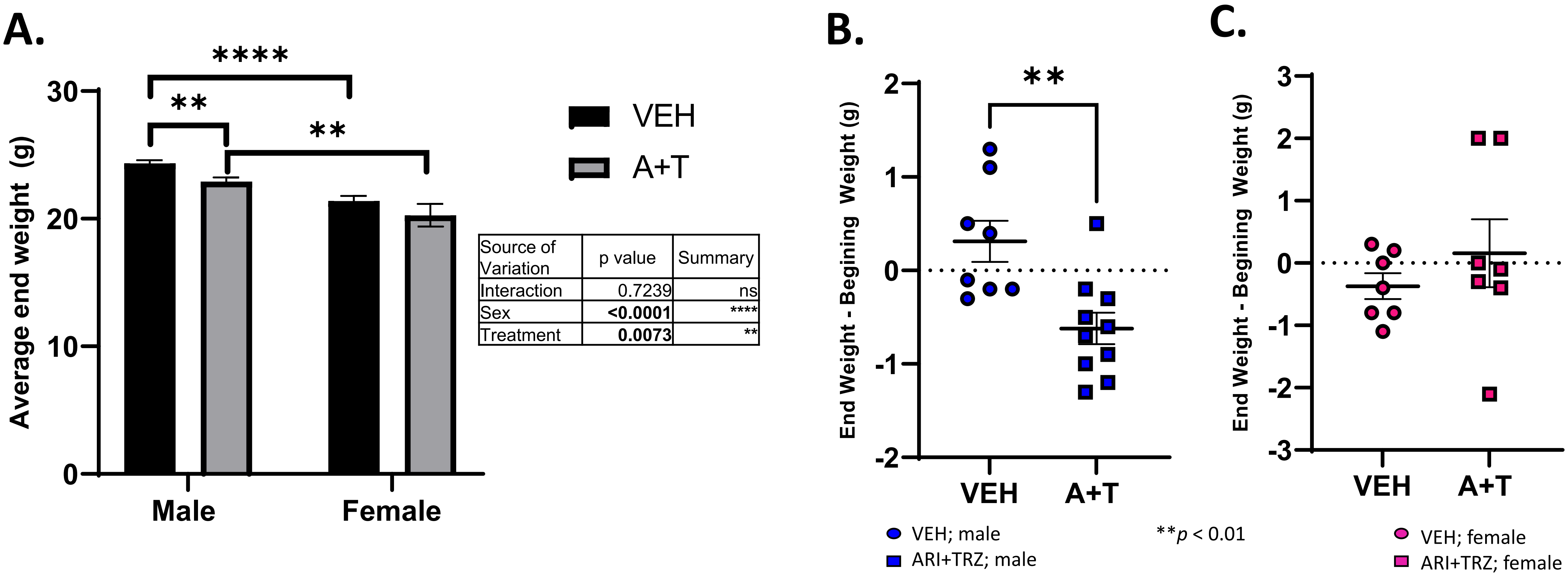

Figure S1. Mouse weights during ARI+TRZ exposure. A) Average body weight of male and female mice at the end of 21-day treatment with ARI (2.5 mg/kg) + TRZ (10 mg/mg). Males VEH average weight (24.34±0.07) vs ARI+TRZ average weight (22.91±0.96) p=0.0041. Females VEH average weight (21.4±0.99) vs A+T average weight (20.27±2.16) p=0.2734. Sex differences for VEH p=<0.0001 and A+T p=0.0059. 2-way ANOVA shows that sex and treatment significantly influenced the body weight (statistics shown in Table S1). B) The change in weight (end of experiment – beginning of experiment) for each male (blue) mouse was graphed. The t-test analysis shows that males exposed to ARI+TRZ lost their weight during treatment. C) The same comparison in female (red) mice did not show difference between control and treated group. **p<0.01; ****p<0.0001. Statistical details are in Table S1.

Table S1.

	VEH			ARI + TRZ				
**A. Mouse Weights**	Mean	SEM	N	Mean	SEM	N	t-test	
male (grams)	24.3375	0.0685	8	22.91	0.9586	10	**0.0041**	**
female (grams)	21.3857	0.9906	7	20.2714	2.1618	7	0.2734	

	VEH			VEH			t-test	
male vs female (grams)	24.3375	0.0685	8	21.3857	0.9906	7	**<0.0001**	****

	ARI + TRZ			ARI + TRZ			t-test	
male vs female (grams)	22.9100	0.9586	10	20.2714	2.1618	7	**0.0059**	**

	VEH			ARI + TRZ				
**B. Delta mouse weight**	Mean	SEM	N	Mean	SEM	N	t-test	
male (grams)	0.3130	0.2200	8	-0.6200	0.1690	10	**0.0035**	**
female (grams)	-0.3710	0.2080	7	0.1570	0.5450	7	0.3830	

Table S1. Mouse weights.

Figure S2.

A.

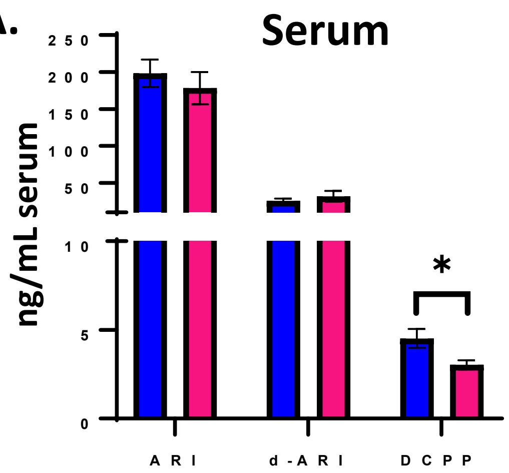

Source of Variation	p value	summary
Interaction	0.5503	ns
Drug and metabolites	**<0.0001**	****
Sex	0.5981	ns

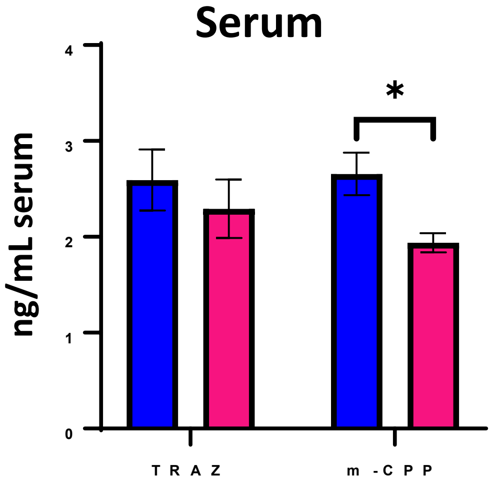

Source of Variation	p value	summary
Interaction	0.4182	ns
Drug and metabolite	0.5726	ns
Sex	0.0597	ns

B.

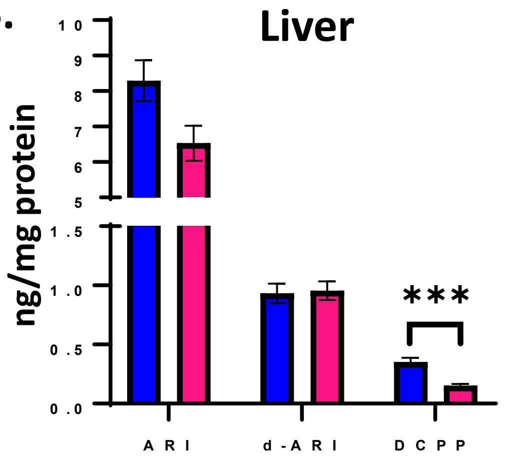

Source of Variation	p value	summary
Interaction	**0.0219**	*
Drug and metabolites	**<0.0001**	****
Sex	**0.0228**	*

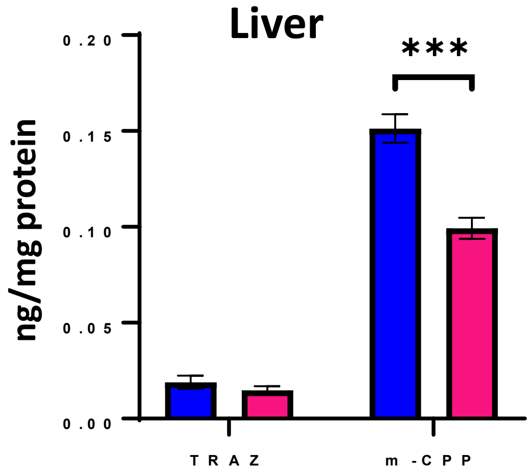

Source of Variation	p value	summary
Interaction	**0.0003**	***
Drug and metabolite	**<0.0001**	****
Sex	**<0.0001**	****

C.

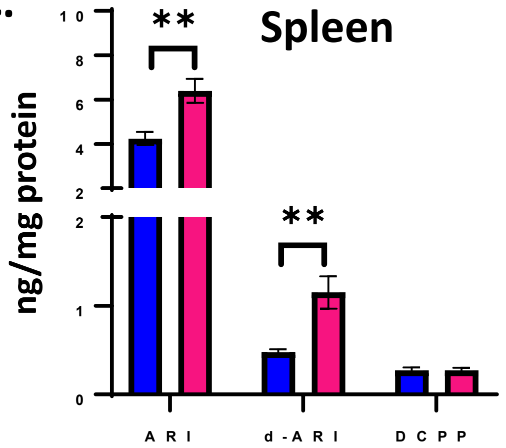

Source of Variation	p value	summary
Interaction	**0.0004**	***
Drug and metabolites	**<0.0001**	****
Sex	**<0.0001**	****

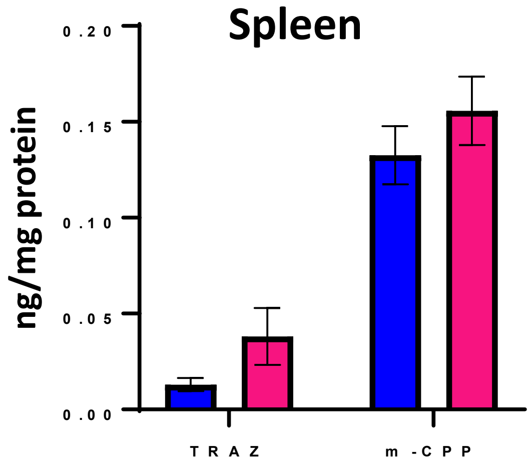

Source of Variation	p value	summary
Interaction	0.9438	ns
Drug and metabolite	**<0.0001**	****
Sex	0.0861	ns

D.

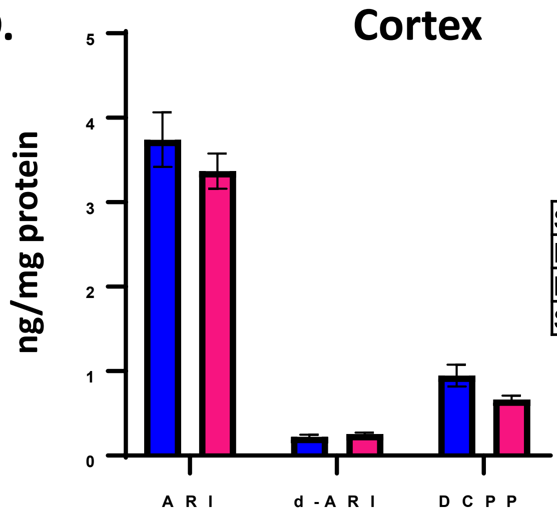

Source of Variation	p value	summary
Interaction	0.5249	ns
Drug and metabolites	**<0.0001**	****
Sex	0.1754	ns

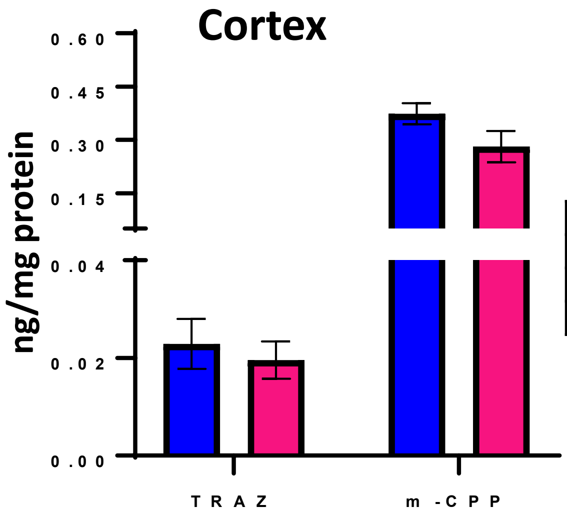

Source of Variation	p value	summary
Interaction	0.1024	ns
Drug and metabolite	**<0.0001**	****
Sex	0.0812	ns

E.

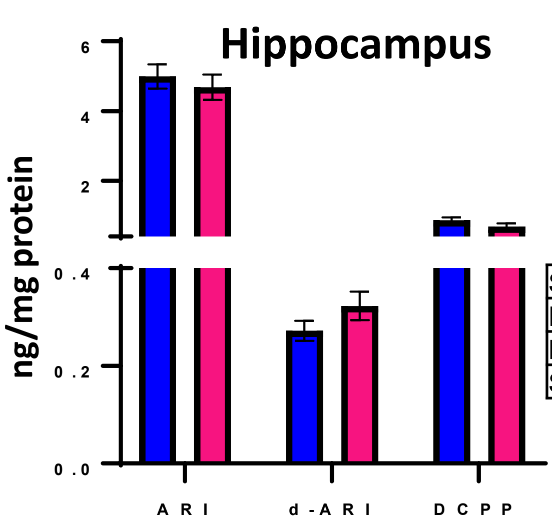

Source of Variation	p value	summary
Interaction	0.6991	ns
Drug and metabolites	**<0.0001**	****
Sex	0.4097	ns

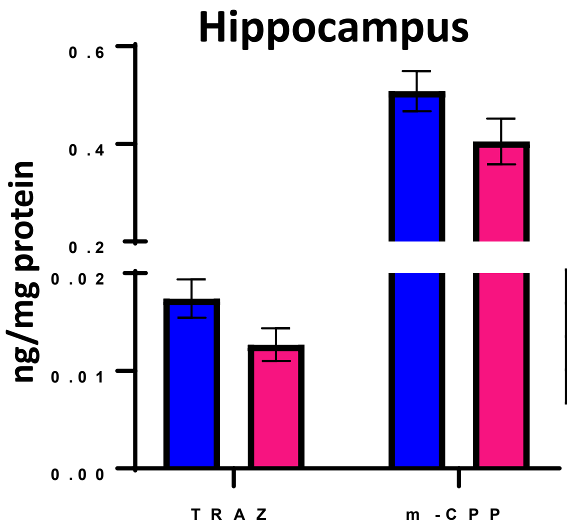

Source of Variation	p value	summary
Interaction	0.1319	ns
Drug and metabolite	**<0.0001**	****
Sex	0.1006	ns

Figure S2. Aripiprazole with its metabolites and trazadone with its metabolite in serum and organs. Average ARI, d-ARI, DCPP TRZ, and m-CPP in males (blue) and females (red) exposed to ARI + TRZ for 21 days with 2-way ANOVA. A) Serum. B) Liver. C) Spleen. D) Cortex. E) Hippocampus. Statistical details are in Table S2.

Table S2.

Supplemental Table 2.

Drug and metabolites	Male			Female			
**A. Serum**	Mean	SEM	N	Mean	SEM	N	t-test
ARI	198.4275	18.51573	5	178.2413	21.83032	5	0.5007
d-ARI	25.9348	2.812522	5	31.75601	7.309506	5	0.4786
DCPP	4.521948	0.532209	5	3.029276	0.266929	5	**0.0365**
TRZ	2.592502	0.317946	5	2.291929	0.305047	5	0.5144
m-CPP	2.656606	0.220812	5	1.938147	0.099891	5	**0.018**
**B. Liver**	Mean	SEM	N	Mean	SEM	N	t-test
ARI	8.29639	0.574082	7	6.532194	0.488176	5	0.0517
d-ARI	0.932552	0.083375	7	0.956004	0.078767	5	0.8483
DCPP	0.353251	0.03387	7	0.153476	0.012421	5	**0.0008**
TRZ	0.018997	0.003472	7	0.014792	0.002055	5	0.3721
m-CPP	0.151327	0.00736	7	0.099285	0.005533	5	**0.0004**
**C. Spleen**	Mean	SEM	N	Mean	SEM	N	t-test
ARI	4.24814	0.295182	7	6.395561	0.542897	5	**0.0038**
d-ARI	0.4768	0.033389	7	1.151228	0.181938	5	**0.0015**
DCPP	0.27137	0.033933	7	0.270561	0.028594	5	0.9866
TRZ	0.012984	0.003413	7	0.038095	0.014815	5	0.0809
m-CPP	0.132545	0.015146	7	0.155746	0.017851	5	0.3454
**D. Cortex**	Mean	SEM	N	Mean	SEM	N	t-test
ARI	3.741342	0.321441	7	3.368334	0.208558	5	0.4640
d-ARI	0.224507	0.023396	7	0.254657	0.019557	5	0.4167
DCPP	0.948305	0.128026	7	0.663347	0.045321	5	0.1340
TRZ	0.02285	0.005115	6	0.019558	0.00381	5	0.6576
m-CPP	0.373692	0.029433	6	0.280919	0.044326	5	0.1195
**E. Hippocampus**	Mean	SEM	N	Mean	SEM	N	t-test
ARI	4.993539	0.347217	7	4.681363	0.363649	5	0.5572
d-ARI	0.271794	0.020703	7	0.322675	0.029317	5	0.1737
DCPP	0.868674	0.077229	7	0.687621	0.091685	5	0.1617
TRZ	0.017395	0.001962	7	0.012677	0.001682	5	0.1152
m-CPP	0.508085	0.04104	7	0.40506	0.046523	5	0.1304

Table S2. Drugs and metabolites in serum and organs.

Figure S3.

A.

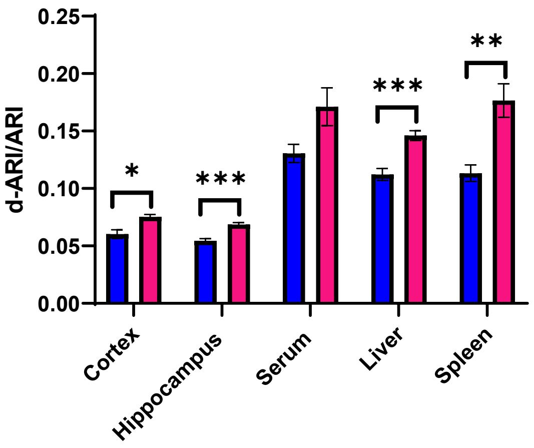

Source of Variation	p value	summary
Interaction	**0.0102**	*
Sex	**<0.0001**	****
Tissue	**<0.0001**	****

B.

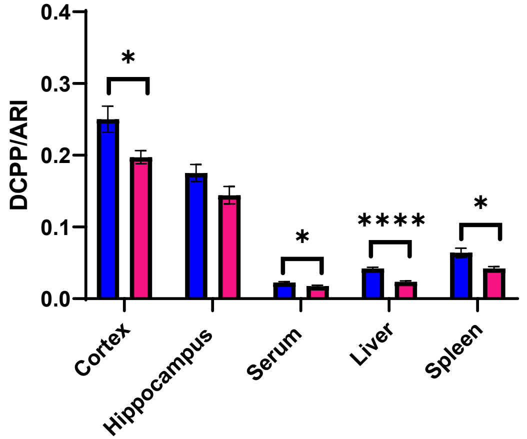

Source of Variation	p value	summary
Interaction	0.1965	ns
Sex	**0.0001**	***
Tissue	**<0.0001**	****

C.

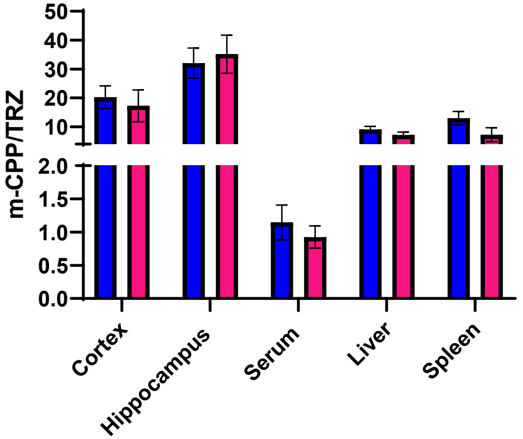

Source of Variation	p value	summary
Interaction	0.7951	ns
Sex	0.5097	ns
Tissue	**<0.0001**	****

Figure S3. Comparison of ARI and TRZ turnover in males and females across tissues. A) D-ARI/ARI ratio is higher in females than in males in all organs. The serum values did not reach statistical significance but showed the same trend as in other organs. B) DCPP/ARI ratio is higher in males than in females. The hippocampus values did not reach statistical significance but showed the same trend as in other organs. C) m-CPP/TRZ ratio is similar in males and females across all organs. Statistical details are in Table S3.

Table S3.

Drug Turnover (au)	Male			Female			
**A. Serum**	Mean	SEM	N	Mean	SEM	N	t-test
d-ARI/ARI	0.1305	0.0079	5	0.1711	0.0164	5	0.0566
DCPP/ARI	0.0226	0.0011	5	0.0174	0.0014	5	**0.0198**
d-ARI+DCPP/ARI	0.1531	0.0074	5	0.1886	0.0153	5	0.0711
m-CPP/TRZ	1.1474	0.2612	5	0.9257	0.1681	5	0.4957
**B. Liver**	Mean	SEM	N	Mean	SEM	N	t-test
d-ARI/ARI	0.1122	0.0052	7	0.1461	0.0042	5	**0.0009**
DCPP/ARI	0.0421	0.0017	7	0.0235	0.0013	5	**<0.0001**
d-ARI+DCPP/ARI	0.1544	0.0051	7	0.1696	0.0055	5	0.0726
m-CPP/TRZ	9.0809	1.1129	7	7.2052	0.9435	5	0.2533
**C. Spleen**	Mean	SEM	N	Mean	SEM	N	t-test
d-ARI/ARI	0.1133	0.0072	7	0.1766	0.0146	5	**0.0016**
DCPP/ARI	0.0640	0.0065	7	0.0422	0.0027	5	**0.0231**
d-ARI+DCPP/ARI	0.1773	0.0095	7	0.2188	0.0144	5	**0.0305**
m-CPP/TRZ	13.0021	2.3042	7	7.2849	2.3885	5	0.1235
**D. Cortex**	Mean	SEM	N	Mean	SEM	N	t-test
d-ARI/ARI	0.0603	0.0038	7	0.0754	0.0020	5	**0.0113**
DCPP/ARI	0.2503	0.0181	7	0.1973	0.0091	5	**0.0441**
d-ARI+DCPP/ARI	0.3106	0.0211	7	0.2727	0.0084	5	0.1798
m-CPP/TRZ	20.2527	3.9339	6	17.2839	5.5079	5	0.6637
**E. Hippocampus**	Mean	SEM	N	Mean	SEM	N	t-test
d-ARI/ARI	0.0544	0.0020	7	0.0686	0.0016	5	**0.0004**
DCPP/ARI	0.1753	0.0120	7	0.1443	0.0122	5	0.1073
d-ARI+DCPP/ARI	0.2297	0.0121	7	0.2129	0.0132	5	0.3753
m-CPP/TRZ	32.0681	5.2047	7	35.1763	6.6177	5	0.7162

Table S3. Drugs turnover in serum and organs.

Figure S4.

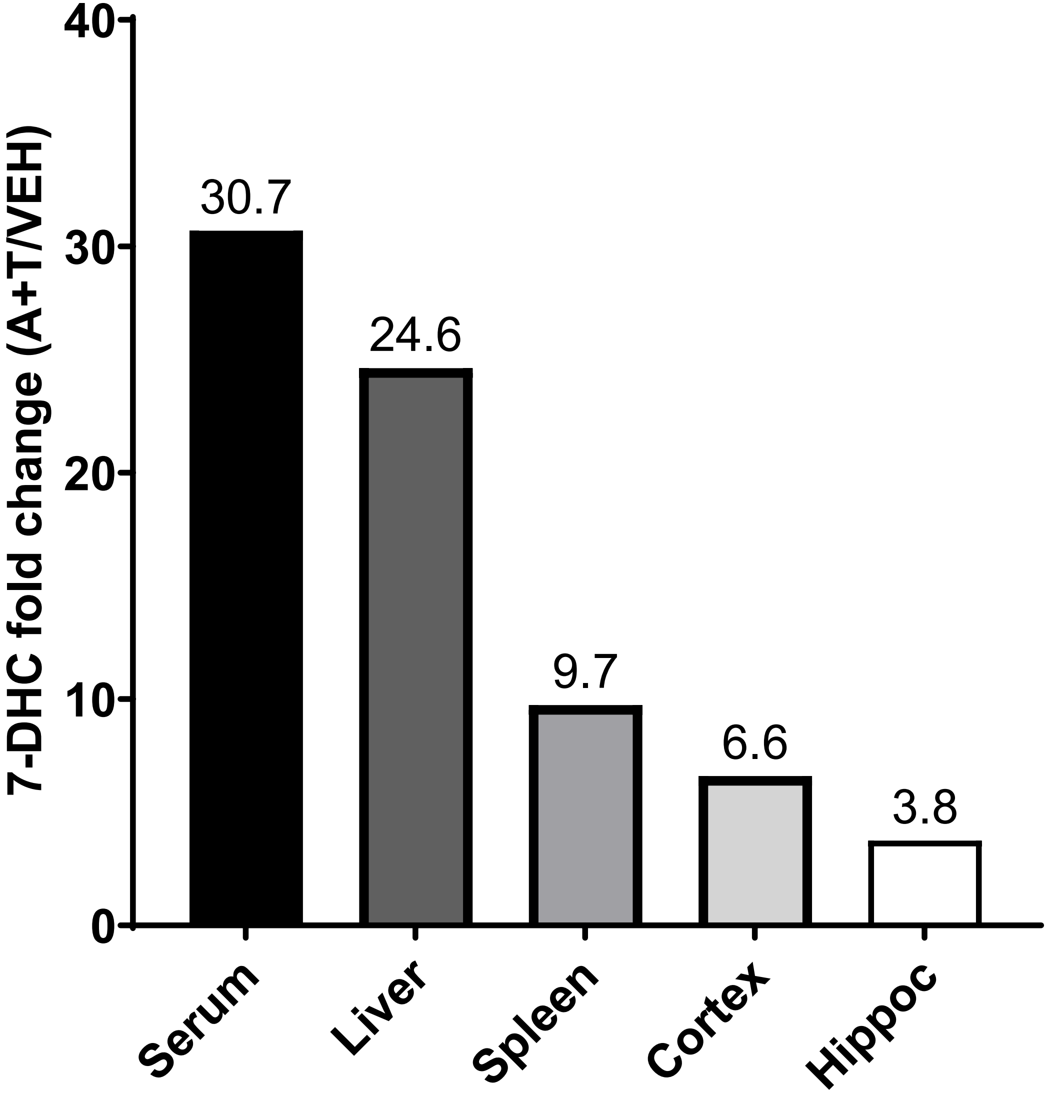

Figure S4. 7-DHC fold increase in response to ARI+TRZ exposure. Compared to the levels of 7-DHC in VEH exposed mice, the levels of 7-DHC in ARI+TRZ exposed mice were significantly increased in all analyzed tissues.

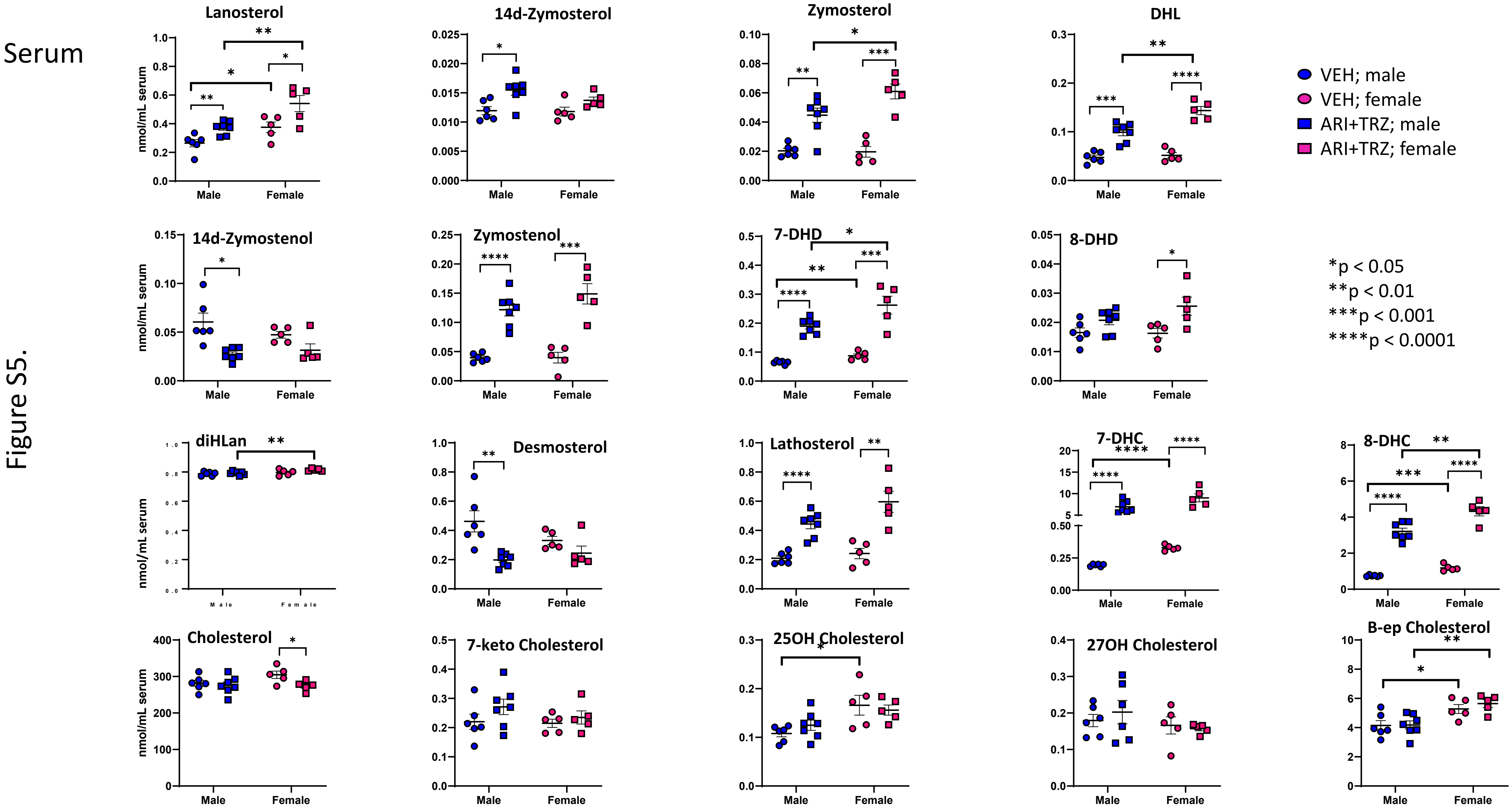

Figure S5. Comparison of sterols and oxysterols between males and females within the serum. Graphs show levels of sterols and oxysterols in males (blue) and females (red) under control (circles) and experimental (squares) conditions. Two-tailed unpaired t-tests were used to determine significance.

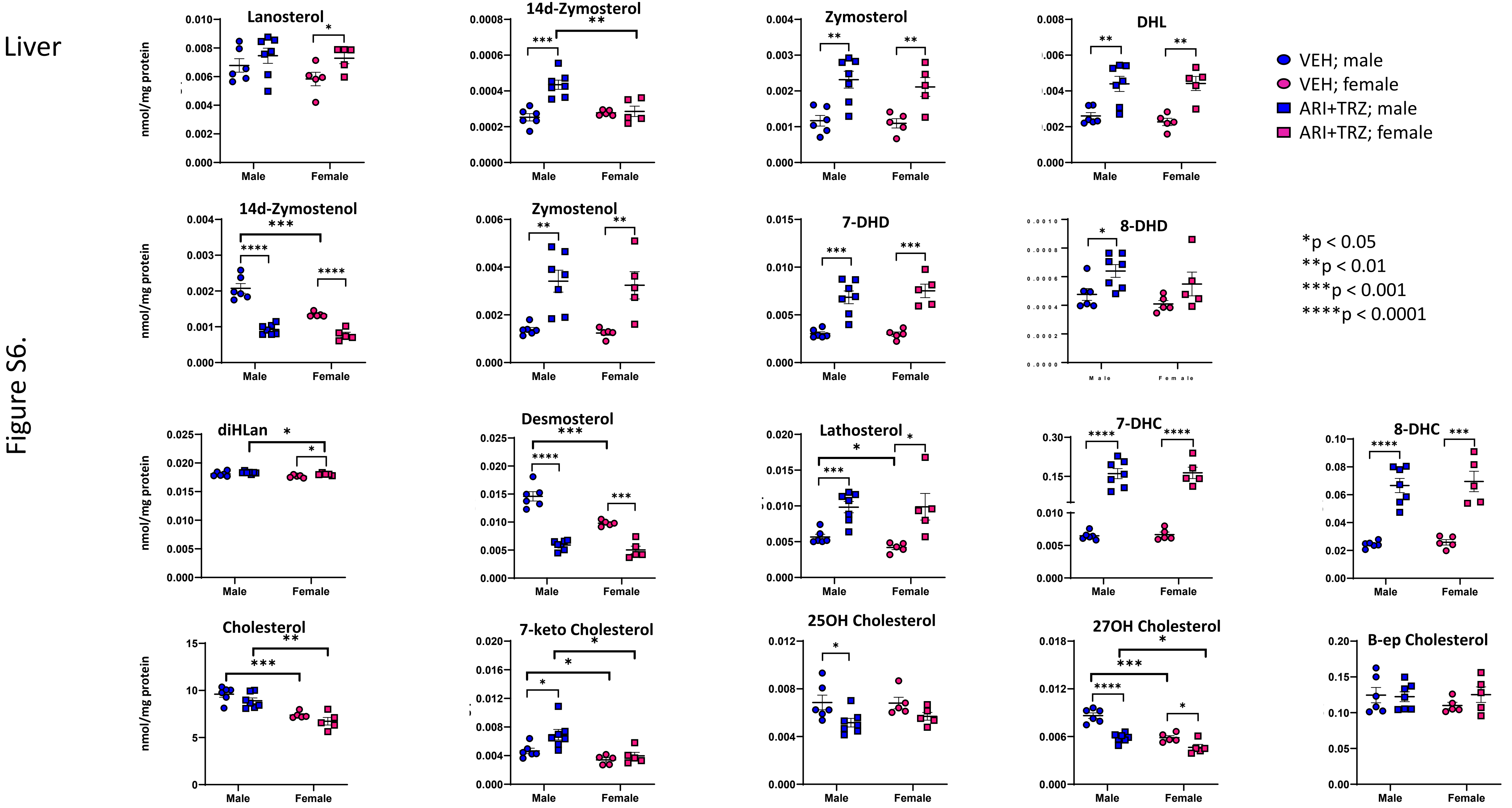

Figure S6. Comparison of sterols and oxysterols between males and females within liver. Graphs show levels of sterols and oxysterols in males (blue) and females (red) under control (circles) and experimental (squares) conditions. Two-tailed unpaired t-tests were used to determine significance.

Figure S7.

Spleen

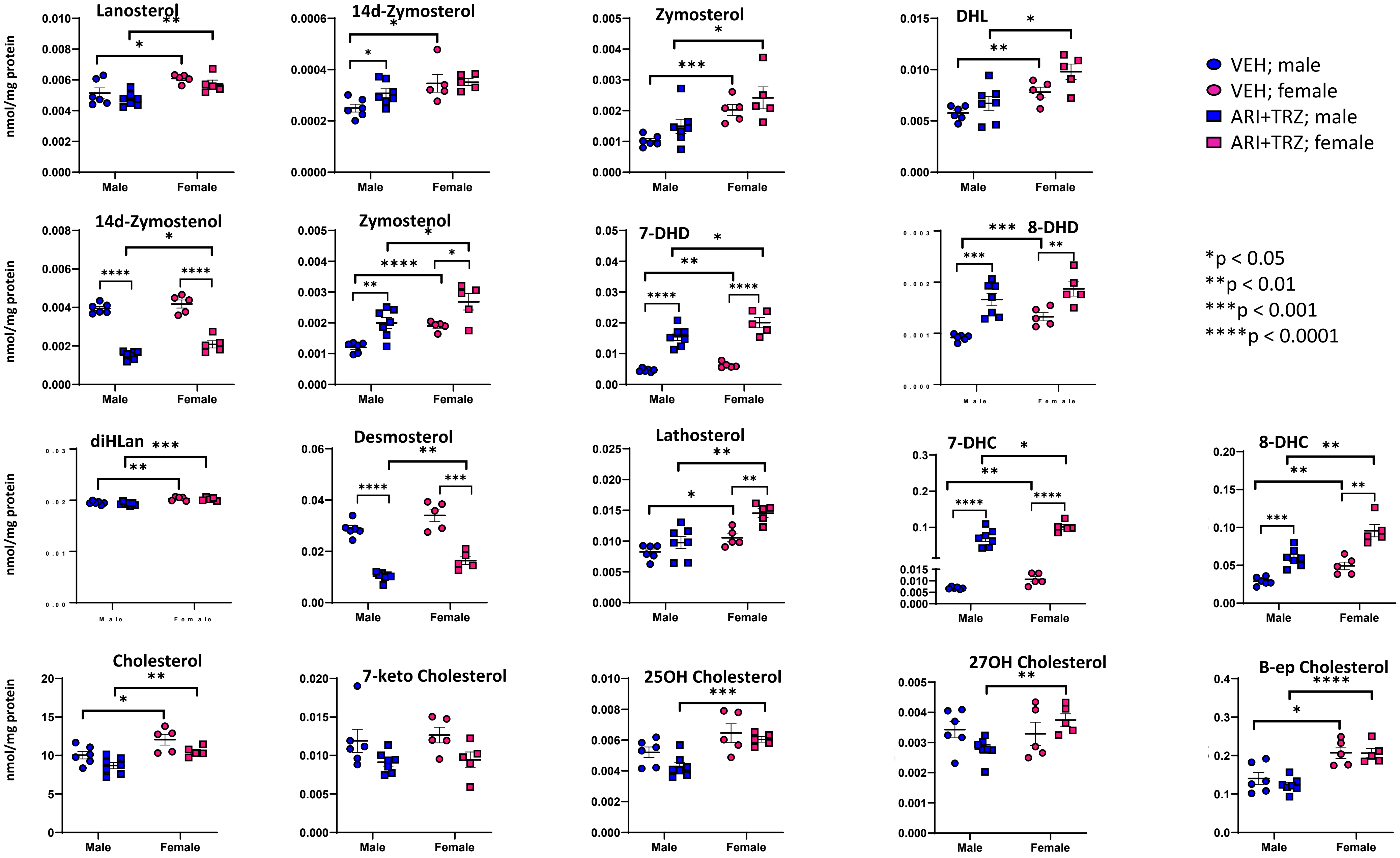

Figure S7. Comparison of sterols and oxysterols between males and females within spleen. Graphs show levels of sterols and oxysterols in males (blue) and females (red) under control (circles) and experimental (squares) conditions. Two-tailed unpaired t-tests were used to determine significance.

Figure S8. Cortex

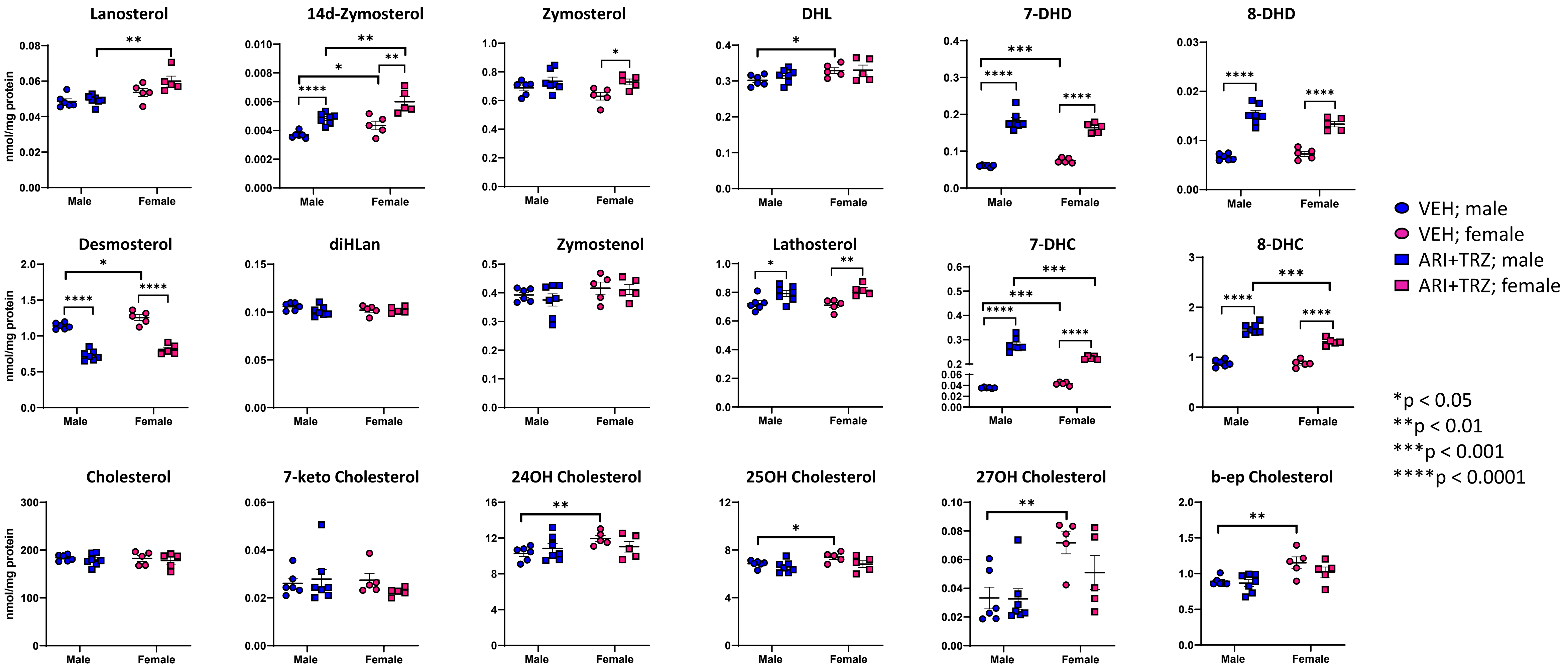

Figure S8. Comparison of sterols and oxysterols between males and females within cortex. Graphs show levels of sterols and oxysterols in males (blue) and females (red) under control (circles) and experimental (squares) conditions. Two-tailed unpaired t-tests were used to determine significance.

Figure S9. Hippocampus

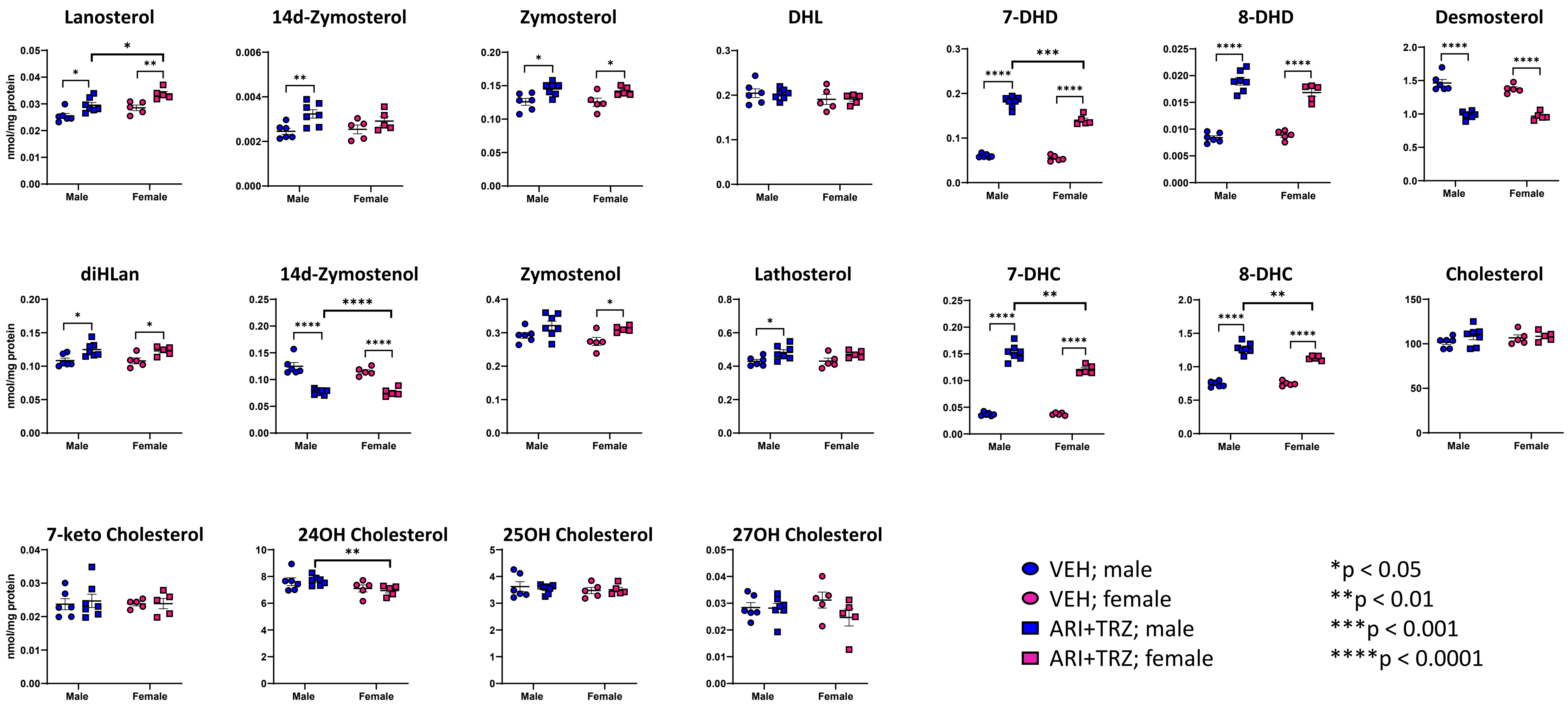

Figure S9. Comparison of sterols and oxysterols between males and females within hippocampus. Graphs show levels of sterols and oxysterols in males (blue) and females (red) under control (circles) and experimental (squares) conditions. Two-tailed unpaired t-tests were used to determine significance.

Figure S10.
Hippocampus

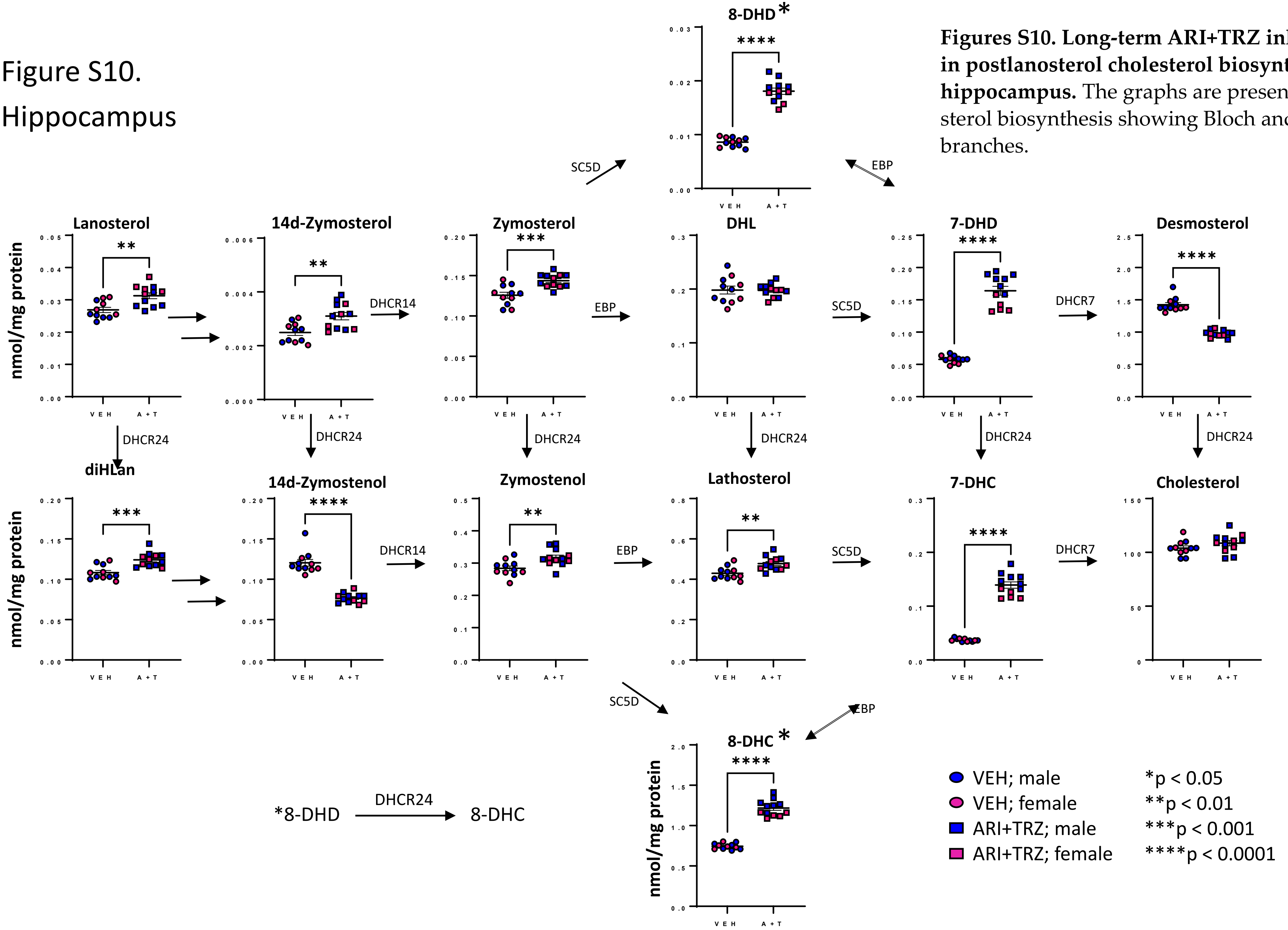

Figures S10. Long-term ARI+TRZ inhibits multiple enzymes in postlanosterol cholesterol biosynthesis pathway within hippocampus. The graphs are presented to coincide with the sterol biosynthesis showing Bloch and Kandutsch-Russell branches.

Table S4.

TABLE S4.

SERUM

	DMG Sterols	Abbr.	VEH mean	VEH SEM	ARI + TRZ mean	ARI + TRZ SEM	t-test p value	
Bloch	Lanosterol	LAN	0.3149	0.0269	0.4425	0.0348	**0.0092**	**
Bloch	14-dehydrozymosterol	14-DZYM	0.0119	0.0005	0.0148	0.0006	**0.0014**	**
Bloch	Zymosterol	ZYM	0.0200	0.0018	0.0515	0.0041	**<0.0001**	****
Bloch	24-dehydrolathosterol	DHL	0.0493	0.0035	0.1177	0.0085	**<0.0001**	****
Bloch	8-dehydrodesmosterol	8-DHD	0.0164	0.0011	0.0227	0.0016	**0.0047**	**
Bloch	7-dehydrodesmosterol	7-DHD	0.0750	0.0047	0.2200	0.0171	**<0.0001**	****
Bloch	Desmosterol	DES	0.4025	0.0447	0.2172	0.0223	**0.0010**	**
Kandutsch Russell	dihydrolanosterol	DIHLAN	0.7923	0.0051	0.8021	0.0046	0.1661	
Kandutsch Russell	14-dehydrozymostenol	14-DZYME	0.0544	0.0054	0.0290	0.0029	**0.0004**	***
Kandutsch Russell	Zymostenol	ZYME	0.03964	0.0042	0.1330	0.0100	**<0.0001**	****
Kandutsch Russell	Lathosterol	LATH	0.2244	0.0180	0.5073	0.0405	**<0.0001**	****
Kandutsch Russell	7-dehyrocholesterol	7-DHC	0.2550	0.0216	7.8250	0.5568	**<0.0001**	****
Kandutsch Russell	8-dehydrocholesterol	8-DHC	0.9467	0.0768	3.6740	0.2192	**<0.0001**	****
	Cholesterol	CHOL	292.0000	7.1940	275.2000	5.7090	0.0790	
Oxysterols	7-keto cholesterol	7-KETO	0.2177	0.0149	0.2560	0.0183	0.1228	
Oxysterols	24OH-cholesterol	24OH-CHOL						
Oxysterols	25OH-cholesterol	25OH-CHOL	0.1343	0.0131	0.1379	0.0086	0.8173	
Oxysterols	27OH-cholesterol	27OH-CHOL	0.1733	0.0135	0.1818	0.0185	0.7146	
Oxysterols	ß-epoxycholesterol	ß-EP CHOL	4.6600	0.2818	4.7910	0.2879	0.7495	

SPLEEN

Abbr.	VEH mean	VEH SEM	ARI + TRZ mean	ARI + TRZ SEM	t-test p value	
LAN	0.0056	0.0002	0.0052	0.0002	0.1866	
14-DZYM	0.0003	0.0000	0.0003	0.0000	0.2203	
ZYM	0.0015	0.0002	0.0019	0.0002	0.1973	
DHL	0.0067	0.0004	0.0080	0.0007	0.1175	
8-DHD	0.0011	0.0001	0.0017	0.0001	**<0.0000**	****
7-DHD	0.0053	0.0003	0.0174	0.0012	**<0.0001**	****
DES	0.0311	0.0015	0.0128	0.0011	**<0.0001**	****
DIHLAN	0.0199	0.0002	0.0197	0.0002	0.4357	
14-DZYME	0.0041	0.0001	0.0018	0.0001	**<0.0001**	****
ZYME	0.0015	0.0001	0.0023	0.0002	**0.0020**	**
LATH	0.0093	0.0005	0.0118	0.0009	**0.0337**	*
7-DHC	0.0086	0.0008	0.0834	0.0073	**<0.0001**	****
8-DHC	0.0382	0.0040	0.0748	0.0067	**0.0002**	***
CHOL	10.9600	0.5030	9.4480	0.3723	**0.0231**	*
7-KETO	0.0123	0.0009	0.0093	0.0005	**0.0073**	**
24OH-CHOL						
25OH-CHOL	0.0058	0.0004	0.0050	0.0003	0.1329	
27OH-CHOL	0.0034	0.0002	0.0032	0.0002	0.5372	
ß-EP CHOL	0.1708	0.0147	0.1579	0.0139	0.5277	

LIVER

Abbr.	VEH mean	VEH SEM	ARI + TRZ mean	ARI + TRZ SEM	t-test p value	
LAN	0.0064	0.0004	0.0074	0.0003	**0.0452**	*
14-DZYM	0.0003	0.0000	0.0004	0.0000	**0.0028**	**
ZYM	0.0011	0.0001	0.0022	0.0002	**<0.0001**	****
DHL	0.0024	0.0001	0.0044	0.0003	**<0.0001**	****
8-DHD	0.0004	0.0000	0.0006	0.0000	**0.0063**	**
7-DHD	0.0030	0.0001	0.0071	0.0005	**<0.0001**	****
DES	0.0124	0.0009	0.0055	0.0003	**<0.0001**	****
DIHLAN	0.0179	0.0001	0.0182	0.0001	**0.0389**	*
14-DZYME	0.0017	0.0001	0.0009	0.0000	**<0.0001**	****
ZYME	0.0013	0.0001	0.0033	0.0003	**<0.0001**	****
LATH	0.0050	0.0003	0.0099	0.0009	**<0.0001**	****
7-DHC	0.0066	0.0002	0.1614	0.0140	**<0.0001**	****
8-DHC	0.0250	0.0010	0.0678	0.0041	**<0.0001**	****
CHOL	8.5990	0.3961	7.9970	0.3965	0.2964	
7-KETO	0.0041	0.0003	0.0057	0.0006	**0.0387**	*
24OH-CHOL						
25OH-CHOL	0.0068	0.0004	0.0054	0.0003	**0.0042**	**
27OH-CHOL	0.0074	0.0005	0.0053	0.0003	**0.0011**	**
ß-EP CHOL	0.1180	0.0063	0.1236	0.0058	0.5162	

CORTEX

Abbr.	VEH mean	VEH SEM	ARI + TRZ mean	ARI + TRZ SEM	t-test p value	
LAN	0.0509	0.0015	0.0538	0.0020	0.2514	
14-DZYM	0.0040	0.0002	0.0053	0.0002	**0.0002**	***
ZYM	0.6622	0.0180	0.7330	0.0178	**0.0109**	*
DHL	0.3145	0.0063	0.3215	0.0072	0.4746	
8-DHD	0.0069	0.0003	0.0145	0.0006	**<0.0001**	****
7-DHD	0.0668	0.0027	0.1746	0.0064	**<0.0001**	****
DES	1.1920	0.0272	0.7646	0.0227	**<0.0001**	****
DIHLAN	0.1041	0.0014	0.1014	0.0013	0.1654	
14-DZYME						
ZYME	0.4034	0.0109	0.3904	0.0147	0.4903	
LATH	0.7172	0.0132	0.8012	0.0138	**0.0003**	***
7-DHC	0.0390	0.0014	0.2576	0.0101	**<0.0001**	****
8-DHC	0.8802	0.0204	1.4650	0.0461	**<0.0001**	****
CHOL	183.2000	3.0090	178.6000	3.8490	0.3616	
7-KETO	0.0267	0.0017	0.0257	0.0024	0.7336	
24OH-CHOL	11.0400	0.3447	10.9200	0.3755	0.8174	
25OH-CHOL	7.0980	0.1348	6.6850	0.1566	0.0609	
27OH-CHOL	0.0508	0.0079	0.0403	0.0067	0.3206	
ß-EP CHOL	1.0100	0.0555	0.9317	0.0451	0.2821	

HIPPOCAMPUS

Abbr.	VEH mean	VEH SEM	ARI + TRZ mean	ARI + TRZ SEM	t-test p value	
LAN	0.0269	0.0008	0.0313	0.0009	**0.0019**	**
14-DZYM	0.0002	0.0001	0.0031	0.0001	**0.0029**	**
ZYM	0.1257	0.0037	0.1438	0.0024	**0.0005**	***
DHL	0.1981	0.0074	0.1976	0.0036	0.9456	
8-DHD	0.0086	0.0003	0.0181	0.0006	**<0.0001**	****
7-DHD	0.0578	0.0018	0.1641	0.0069	**<0.0001**	****
DES	1.4240	0.0327	0.9781	0.0155	**<0.0001**	****
DIHLAN	0.1082	0.0027	0.1241	0.0025	**0.0003**	***
14-DZYME	0.1204	0.0042	0.0769	0.0002	**<0.0001**	****
ZYME	0.2838	0.0074	0.3171	0.0077	**0.0052**	**
LATH	0.4296	0.0096	0.4780	0.0099	**0.0022**	**
7-DHC	0.0371	0.0008	0.1393	0.0059	**<0.0001**	****
8-DHC	0.7439	0.0116	1.2170	0.0284	**<0.0001**	****
CHOL	104.0000	2.1100	108.5000	2.5010	0.1800	
7-KETO	0.0238	0.0009	0.0244	0.0013	0.7163	
24OH-CHOL	7.3830	0.2066	7.3750	0.1477	0.9747	
25OH-CHOL	3.5570	0.1101	3.5240	0.0496	0.7817	
27OH-CHOL	0.0297	0.0017	0.0267	0.0017	0.2238	
ß-EP CHOL						

these numbers are males and females combined (NOT SEPARATE)

Table S4. Sterols and oxysterol levels (Mean±SEM) in serum, and organs in VEH and ARI+TRZ exposed mice.

Table S5.

			SERUM									
			VEH		VEH			ARI + TRZ		ARI + TRZ		
			SEX		SEX			SEX		SEX		
			Male		Female		VEH	Male		Female		A+T
	DMG Sterols	Abbr.	mean	SEM	mean	SEM	p value	mean	SEM	mean	SEM	p value
Bloch	Lanosterol	LAN	0.2645	0.0254	0.3753	0.0364	**0.0306**	0.3721	0.0176	0.5412	0.0562	**0.0079**
	14-dehydrozymosterol	14-DZYM	0.0120	0.0007	0.0118	0.0008	0.8825	0.0155	0.0009	0.0137	0.0006	0.1567
	Zymosterol	ZYM	0.0203	0.0017	0.0196	0.0036	0.8555	0.0447	0.0048	0.0610	0.0051	**0.0469**
	24-dehydrolathosterol	DHL	0.0474	0.0047	0.0516	0.0058	0.5776	0.0991	0.0073	0.1436	0.0085	**0.0027**
	8-dehydrodesmosterol	8-DHD	0.0165	0.0016	0.0163	0.0016	0.9160	0.0207	0.0015	0.0255	0.0031	0.1550
	7-dehydrodesmosterol	7-DHD	0.0647	0.0024	0.0874	0.0066	**0.0068**	0.1898	0.0100	0.2622	0.0309	**0.0284**
	Desmosterol	DES	0.4619	0.0727	0.3312	0.0272	0.1544	0.1977	0.0171	0.2447	0.0485	0.3220
Kandutsch Russell	dihydrolanosterol	DIHLAN	0.7880	0.0057	0.7972	0.0092	0.4045	0.7928	0.0048	0.8152	0.0041	**0.0073**
	14-dehydrozymostenol	14-DZYME	0.0604	0.0092	0.0473	0.0034	0.2463	0.0272	0.0024	0.0315	0.0064	0.4973
	Zymostenol	ZYME	0.0395	0.0029	0.0398	0.0092	0.9793	0.1217	0.0108	0.1489	0.0174	0.1913
	Lathosterol	LATH	0.2097	0.0161	0.2420	0.0354	0.4013	0.4439	0.0325	0.5962	0.0723	0.0588
	7-dehyrocholesterol	7-DHC	0.1940	0.0040	0.3281	0.0098	**<0.0001**	6.9694	0.5152	9.0219	0.9349	0.6510
	8-dehydrocholesterol	8-DHC	0.7491	0.0173	1.1838	0.0787	**0.0002**	3.2080	0.1825	4.3260	0.2547	**0.0043**
	Cholesterol	CHOL	281.4323	8.5194	304.6943	10.0970	0.1097	276.1044	9.1612	273.9122	6.1786	0.8601
Oxysterols	7-keto cholesterol	7-KETO	0.2200	0.0260	0.2149	0.0142	0.8735	0.2710	0.0268	0.2349	0.0224	0.3541
	24OH-cholesterol	24OH-CHOL										
	25OH-cholesterol	25OH-CHOL	0.1078	0.0068	0.1660	0.0203	**0.0165**	0.1249	0.0105	0.1560	0.0104	0.0699
	27OH-cholesterol	27OH-CHOL	0.1793	0.0169	0.1661	0.0235	0.6510	0.2024	0.0323	0.1572	0.0059	0.2425
	ß-epoxycholesterol	ß-EP CHOL	4.1464	0.3352	5.2763	0.3066	**0.0372**	4.1819	0.2798	5.6430	0.2668	**0.0046**

	SPLEEN									
	VEH		VEH			ARI + TRZ		ARI + TRZ		
	SEX		SEX			SEX		SEX		
	Male		Female		VEH	Male		Female		A+T
Abbr.	mean	SEM	mean	SEM	p value	mean	SEM	mean	SEM	p value
LAN	0.0051	0.0003	0.0061	0.0001	**0.0382**	0.0047	0.0002	0.0057	0.0003	**0.0079**
14-DZYM	0.0003	0.0000	0.0003	0.0000	**0.0232**	0.0003	0.0000	0.0004	0.0000	0.0966
ZYM	0.0010	0.0001	0.0020	0.0002	**0.0003**	0.0015	0.0002	0.0024	0.0004	**0.0459**
DHL	0.0058	0.0003	0.0078	0.0005	**0.0044**	0.0067	0.0007	0.0098	0.0008	**0.0124**
8-DHD	0.0009	0.0000	0.0013	0.0001	**0.0005**	0.0017	0.0001	0.0019	0.0001	0.2958
7-DHD	0.0046	0.0002	0.0062	0.0004	**0.0048**	0.0155	0.0012	0.0201	0.0017	**0.0464**
DES	0.0287	0.0013	0.0340	0.0024	0.0732	0.0103	0.0006	0.0164	0.0015	**0.0019**
DIHLAN	0.0195	0.0001	0.0203	0.0002	**0.0045**	0.0193	0.0001	0.0202	0.0001	**0.0007**
14-DZYME	0.0039	0.0001	0.0042	0.0002	0.3260	0.0015	0.0001	0.0021	0.0002	**0.0110**
ZYME	0.0012	0.0001	0.0019	0.0001	**<0.0001**	0.0020	0.0002	0.0027	0.0003	**0.0486**
LATH	0.0082	0.0005	0.0105	0.0006	**0.0171**	0.0098	0.0010	0.0146	0.0007	**0.0039**
7-DHC	0.0068	0.0002	0.0107	0.0011	**0.0054**	0.0704	0.0090	0.1015	0.0064	**0.0278**
8-DHC	0.0290	0.0021	0.0492	0.0052	**0.0038**	0.0599	0.0045	0.0956	0.0081	**0.0020**
CHOL	10.0537	0.4909	12.0562	0.6952	**0.0390**	8.6837	0.4030	10.5178	0.2883	**0.0068**
7-KETO	0.0119	0.0015	0.0127	0.0010	0.7016	0.0091	0.0005	0.0094	0.0010	0.7875
24OH-CHOL										
25OH-CHOL	0.0052	0.0003	0.0065	0.0006	0.0911	0.0043	0.0003	0.0060	0.0002	**0.0005**
27OH-CHOL	0.0034	0.0003	0.0033	0.0004	0.7681	0.0028	0.0001	0.0037	0.0002	**0.0026**
ß-EP CHOL	0.1406	0.0155	0.2071	0.0149	**0.0137**	0.1231	0.0072	0.2066	0.0119	**<0.0001**

	LIVER									
	VEH		VEH			ARI + TRZ		ARI + TRZ		
	SEX		SEX			SEX		SEX		
	Male		Female		VEH	Male		Female		A+T
Abbr.	mean	SEM	mean	SEM	p value	mean	SEM	mean	SEM	p value
LAN	0.0068	0.0005	0.0058	0.0005	0.1943	0.0075	0.0005	0.0073	0.0004	0.8159
14-DZYM	0.0003	0.0000	0.0003	0.0000	0.3224	0.0004	0.0000	0.0003	0.0000	**0.0036**
ZYM	0.0012	0.0001	0.0011	0.0001	0.7265	0.0023	0.0002	0.0021	0.0003	0.5810
DHL	0.0026	0.0002	0.0023	0.0002	0.2657	0.0044	0.0004	0.0044	0.0004	0.9694
8-DHD	0.0005	0.0000	0.0004	0.0000	0.2213	**0.0006**	**0.0000**	**0.0005**	**0.0001**	0.3208
7-DHD	0.0030	0.0002	0.0030	0.0002	0.8432	0.0068	0.0007	0.0075	0.0007	0.5126
DES	0.0146	0.0008	0.0098	0.0002	**0.0007**	0.0059	0.0003	0.0050	0.0007	0.2239
DIHLAN	0.0181	0.0002	0.0177	0.0001	0.0793	**0.0183**	**0.0001**	**0.0180**	**0.0001**	**0.0336**
14-DZYME	0.0021	0.0001	0.0013	0.0000	**0.0009**	0.0009	0.0001	0.0008	0.0001	0.1174
ZYME	0.0014	0.0001	0.0012	0.0001	0.3201	0.0034	0.0005	0.0032	0.0006	0.8175
LATH	0.0057	0.0004	0.0042	0.0003	**0.0185**	0.0098	0.0008	0.0099	0.0019	0.9784
7-DHC	0.0065	0.0002	0.0067	0.0004	0.6749	0.1601	0.0195	0.1631	0.0220	0.9229
8-DHC	0.0243	0.0010	0.0258	0.0020	0.4868	0.0666	0.0051	0.0695	0.0073	0.7446
CHOL	9.6096	0.3305	7.3852	0.1565	**0.0003**	8.9033	0.3001	6.7287	0.3982	**0.0012**
7-KETO	0.0047	0.0004	0.0034	0.0003	**0.0344**	0.0069	0.0007	0.0040	0.0005	**0.0128**
24OH-CHOL										
25OH-CHOL	0.0069	0.0006	0.0068	0.0005	0.9531	0.0052	0.0004	0.0057	0.0003	0.3184
27OH-CHOL	0.0086	0.0003	0.0059	0.0002	**0.0002**	0.0058	0.0002	0.0046	0.0004	**0.0119**
ß-EP CHOL	0.1246	0.0106	0.1100	0.0045	0.2722	0.1224	0.0069	0.1253	0.0108	0.8159

			CORTEX									
			VEH		VEH			ARI + TRZ		ARI + TRZ		
			SEX		SEX			SEX		SEX		
			Male		Female		VEH	Male		Female		A+T
	DMG Sterols	Abbr.	mean	SEM	mean	SEM	p value	mean	SEM	mean	SEM	p value
Bloch	Lanosterol	LAN	0.0486	0.0015	0.0536	0.0022	0.0846	0.0494	0.0010	0.0600	0.0028	**0.0022**
	14-dehydrozymosterol	14-DZYM	0.0037	0.0001	0.0043	0.0003	**0.0476**	0.0048	0.0001	0.0060	0.0004	**0.0073**
	Zymosterol	ZYM	0.6886	0.0203	0.6307	0.0267	0.1125	0.7353	0.0281	0.7299	0.0203	0.8885
	24-dehydrolathosterol	DHL	0.0070	0.0003	0.0070	0.0005	**0.0256**	0.0150	0.0007	0.0130	0.0006	0.3000
	8-dehydrodesmosterol	8-DHD	0.0066	0.0003	0.0073	0.0005	0.2560	0.0153	0.0007	0.0133	0.0006	0.0783
	7-dehydrodesmosterol	7-DHD	0.0603	0.0010	0.0747	0.0030	**0.0008**	0.1824	0.0093	0.1637	0.0059	0.1548
	Desmosterol	DES	1.1392	0.0174	1.2561	0.0420	**0.0223**	0.7297	0.0259	0.8135	0.0310	0.0641
Kandutsch Russell	dihydrolanosterol	DIHLAN	0.3023	0.0062	0.3291	0.0081	0.1866	0.3149	0.0074	0.3307	0.0137	0.6843
	14-dehydrozymostenol	14-DZYME										
	Zymostenol	ZYME	0.3926	0.0093	0.4164	0.0212	0.3015	0.3751	0.0212	0.4118	0.0169	
	Lathosterol	LATH	0.7225	0.0200	0.7108	0.0182	0.6809	0.7914	0.0205	0.8148	0.0169	0.4281
	7-dehyrocholesterol	7-DHC	0.0356	0.0004	0.0431	0.0015	**0.0004**	0.2804	0.0103	0.2256	0.0033	**0.0015**
	8-dehydrocholesterol	8-DHC	0.8833	0.0288	0.8765	0.0323	0.8774	1.5750	0.0372	1.3115	0.0314	**0.0005**
	Cholesterol	CHOL	183.7624	2.7001	182.6276	6.2187	0.8623	179.1427	4.6799	177.9157	7.1826	0.8837
Oxysterols	7-keto cholesterol	7-KETO	0.0261	0.0021	0.0274	0.0029	0.7118	0.0279	0.0040	0.0226	0.0008	0.3012
	24OH-cholesterol	24OH-CHOL	10.2862	0.3264	11.9475	0.3425	**0.0068**	10.8461	0.5226	11.0270	0.5935	0.0741
	25OH-cholesterol	25OH-CHOL	6.8419	0.1186	7.4049	0.1887	**0.0278**	6.5908	0.1886	6.8170	0.2822	0.5027
	27OH-cholesterol	27OH-CHOL	0.0333	0.0075	0.0717	0.0077	**0.0063**	0.0326	0.0071	0.0510	0.0118	0.1868
	ß-epoxycholesterol	ß-EP CHOL	0.8922	0.0248	1.1515	0.0827	**0.0098**	0.8677	0.0486	1.0214	0.0706	0.0924

	HIPPOCAMPUS									
	Control		Control			ARI + TRZ		ARI + TRZ		
	SEX		SEX			SEX		SEX		
	Male		Female		VEH	Male		Female		A+T
Abbr.	mean	SEM	mean	SEM	p value	mean	SEM	mean	SEM	p value
LAN	0.0255	0.0009	0.0285	0.0010	0.0584	0.0295	0.0010	0.0338	0.0009	**0.0146**
14-DZYM	0.0025	0.0001	0.0025	0.0002	0.7080	0.0032	0.0002	0.0029	0.0002	0.2837
ZYM	0.1259	0.0052	0.1254	0.0061	0.9466	0.1451	0.0037	0.1419	0.0029	0.5395
DHL	0.2044	0.0098	0.1906	0.0114	0.3773	0.2021	0.0045	0.1913	0.0050	0.1452
8-DHD	0.0084	0.0004	0.0089	0.0004	0.3922	0.0190	0.0007	0.0168	0.0007	0.0686
7-DHD	0.0603	0.0017	0.0548	0.0029	0.1257	0.1811	0.0047	0.1402	0.0048	**0.0001**
DES	1.4634	0.0518	1.3766	0.0288	0.2007	0.9853	0.0202	0.9681	0.0262	0.6081
DIHLAN	0.1085	0.0037	0.1079	0.0044	0.9286	0.1250	0.0040	0.1228	0.0029	0.6858
14-DZYME	0.1248	0.0068	0.1152	0.0035	0.8793	0.0771	0.0018	0.0765	0.0035	**<0.0001**
ZYME	0.2921	0.0085	0.2740	0.0121	0.2406	0.3214	0.0130	0.3112	0.0040	0.5389
LATH	0.4290	0.0110	0.4303	0.0182	0.9520	0.4825	0.0160	0.4717	0.0096	0.6165
7-DHC	0.0370	0.0014	0.0372	0.0011	0.9235	0.1526	0.0057	0.1207	0.0036	**0.0016**
8-DHC	0.7409	0.0170	0.7475	0.0156	0.7845	1.2782	0.0307	1.1314	0.0144	**0.0035**
CHOL	101.6736	2.5296	106.6869	3.3855	0.2570	#######	4.0847	108.4364	2.5002	0.9754
7-KETO	0.0237	0.0016	0.0238	0.0006	0.9614	0.0247	0.0020	0.0239	0.0015	0.7685
24OH-CHOL	7.6125	0.2944	7.1081	0.2649	0.2432	7.6809	0.1303	6.9472	0.1726	**0.0061**
25OH-CHOL	3.6248	0.1826	3.4751	0.1169	0.5274	3.5358	0.0638	3.5070	0.0869	0.7900
27OH-CHOL	0.0284	0.0018	0.0312	0.0030	0.4290	0.0282	0.0017	0.0247	0.0032	0.3206
ß-EP CHOL										

Table S5. Sterols and oxysterol levels (Mean±SEM) in serum and organs in male and female VEH and ARI+TRZ exposed mice.

Table S5.

SERUM — p value, VEH vs ARI+TRZ

	DMG Sterols		Males		Females	
Bloch	Lanosterol	LAN	**0.0044**	**	**0.0383**	*
	14-dehydrozymosterol	14-DZYM	**0.0104**	*	0.0756	
	Zymosterol	ZYM	**0.0010**	**	**0.0002**	***
	24-dehydrolathosterol	DHL	**0.0001**	***	**<0.0001**	****
	8-dehydrodesmosterol	8-DHD	0.0817		**0.0289**	*
	7-dehydrodesmosterol	7-DHD	**<0.0001**	****	**0.0006**	***
	Desmosterol	DES	**0.0029**	**	0.1585	
Kandutsch Russell	dihydrolanosterol	DIHLAN	0.5432		0.1130	
	14-dehydrozymostenol	14-DZYME	**0.0031**	**	0.0619	
	Zymostenol	ZYME	**<0.0001**	****	**0.0005**	***
	Lathosterol	LATH	**<0.0001**	****	**0.0023**	**
	7-dehyrocholesterol	7-DHC	**<0.0001**	****	**<0.0001**	****
	8-dehydrocholesterol	8-DHC	**<0.0001**	****	**<0.0001**	****
	Cholesterol	CHOL	0.6821		**0.0316**	*
Oxysterols	7-keto cholesterol	7-KETO	0.2030		0.4716	
	24OH-cholesterol	24OH-CHOL				
	25OH-cholesterol	25OH-CHOL	0.2170		0.6723	
	27OH-cholesterol	27OH-CHOL	0.5418		0.7223	
	ß-epoxycholesterol	ß-EP CHOL	0.9362		0.3933	

t-test here comparing Male VEH vs Male ARI+TR and Female VEH vs Female ARI+TRZ

SPLEEN — p value, VEH vs ARI+TRZ

DMG Sterols	Males		Females	
LAN	0.2856		0.2478	
14-DZYM	**0.0313**	*	0.8996	
ZYM	0.0979		0.3563	
DHL	0.2457		0.0584	
8-DHD	**0.0002**	***	**0.0094**	**
7-DHD	**<0.0001**	****	**<0.0001**	****
DES	**<0.0001**	****	**0.0003**	***
DIHLAN	0.2767		0.7318	
14-DZYME	**<0.0001**	****	**<0.0001**	****
ZYME	**0.0024**	**	**0.0213**	*
LATH	0.2013		**0.0027**	**
7-DHC	**<0.0001**	****	**<0.0001**	****
8-DHC	**0.0001**	***	**0.0013**	**
CHOL	0.052		0.0752	
7-KETO	0.0888		0.0568	
24OH-CHOL				
25OH-CHOL	0.0573		0.5319	
27OH-CHOL	0.0527		0.3227	
ß-EP CHOL	0.3049		0.9782	

LIVER — p value, VEH vs ARI+TRZ

DMG Sterols	Males		Females	
LAN	0.3689		**0.0443**	*
14-DZYM	**0.0002**	***	0.7814	
ZYM	**0.0023**	**	**0.0084**	**
DHL	**0.0038**	**	**0.0012**	**
8-DHD	**0.0219**	*	0.1488	
7-DHD	**0.0004**	***	**0.0003**	***
DES	**<0.0001**	****	**0.0002**	***
DIHLAN	0.2216		**0.0348**	*
14-DZYME	**<0.0001**	****	**<0.0001**	****
ZYME	**0.0020**	**	**0.0086**	**
LATH	**0.0010**	***	**0.0165**	*
7-DHC	**<0.0001**	****	**<0.0001**	****
8-DHC	**<0.0001**	****	**0.0004**	***
CHOL	0.1413		0.1635	
7-KETO	**0.0255**	*	0.3572	
24OH-CHOL				
25OH-CHOL	**0.0318**	*	0.0949	
27OH-CHOL	**<0.0001**	****	**0.0265**	*
ß-EP CHOL	0.8614		0.2290	

CORTEX — p value, VEH vs ARI+TRZ

DMG Sterols	Males		Females	
LAN	0.6450		0.1099	
14-DZYM	**<0.0001**	****	**0.0081**	**
ZYM	0.2187		**0.0183**	
DHL	0.2237		0.9234	
8-DHD	**<0.0001**	****	**<0.0001**	****
7-DHD	**<0.0001**	****	**<0.0001**	****
DES	**<0.0001**	****	**<0.0001**	****
DIHLAN	0.0865		0.9878	
14-DZYME				
ZYME	0.4914		0.8678	
LATH	**0.0363**	*	**0.0030**	**
7-DHC	**<0.0001**	****	**<0.0001**	****
8-DHC	**<0.0001**	****	**<0.0001**	****
CHOL	0.4317		0.6333	
7-KETO	0.7164		0.1415	
24OH-CHOL	0.4020		0.2160	
25OH-CHOL	0.3028		0.1215	
27OH-CHOL	0.9488		0.1789	
ß-EP CHOL	0.6782		0.2660	

HIP — p value, VEH vs ARI+TRZ

DMG Sterols	Males		Females	
LAN	**0.0156**	*	**0.0054**	**
14-DZYM	**0.0078**	**	0.2151	
ZYM	**0.0105**	*	**0.039**	*
DHL	0.8234		0.9566	
8-DHD	**<0.0001**	****	**<0.0001**	****
7-DHD	**<0.0001**	****	**<0.0001**	****
DES	**<0.0001**	****	**<0.0001**	****
DIHLAN	**0.0121**	*	**0.0224**	*
14-DZYME	**<0.0001**	****	**<0.0001**	****
ZYME	0.0974		**0.0195**	*
LATH	**0.0226**	*	0.0784	
7-DHC	**<0.0001**	****	**<0.0001**	****
8-DHC	**<0.0001**	****	**<0.0001**	****
CHOL	0.1941		0.6886	
7-KETO	0.7205		0.9739	
24OH-CHOL	0.8268		0.6244	
25OH-CHOL	0.6335		0.8319	
27OH-CHOL	0.9336		0.1760	
ß-EP CHOL				

Table S5. Sterols and oxysterol levels (Mean±SEM) in serum and organs in male and female VEH and ARI+TRZ exposed mice.
